# Exopolysaccharide from *Cryptococcus heimaeyensis* S20 induces autophagic cell death in non‐small cell lung cancer cells via ROS/p38 and ROS/ERK signalling

**DOI:** 10.1111/cpr.12869

**Published:** 2020-06-29

**Authors:** Yao Hao, Yao Huang, Jingyi Chen, Jiadai Li, Yuncong Yuan, Mingzhen Wang, Lingling Han, Xiu Xin, Hailong Wang, Danqing Lin, Fang Peng, Fang Yu, Congyi Zheng, Chao Shen

**Affiliations:** ^1^ State Key Laboratory of Virology College of Life Sciences Wuhan University Wuhan China; ^2^ College of Life Sciences Wuhan University Wuhan China; ^3^ Department of Pathology Zhongnan Hospital Wuhan University; ^4^ China Center for Type Culture Collection Wuhan University Wuhan China; ^5^ Institute of Pathogenic Microorganism and College of Bioscience and Engineering Jiangxi Agricultural University Nanchang China

**Keywords:** Autophagic cell death, CHEPS, NSCLC, p38/ERK MAPK, ROS, S and G2/M arrest

## Abstract

**Objectives:**

*Cryptococcus heimaeyensis* S20 is found in Antarctica and can produce exopolysaccharides (CHEPS). Here, we explore the anti‐tumour effects of CHEPS on non‐small cell lung cancer (NSCLC).

**Materials and methods:**

Cell viability was assessed by CCK8 and colony formation assays. Flow cytometry was used to analyse the cell cycle, cell apoptosis and reactive oxygen species (ROS). Cell autophagy was detected by EGFP‐LC3 puncta assay, Lyso‐Tracker Red staining and transmission electron microscopy. mRNA and protein levels were analysed by qRT‐PCR and Western blot. Related mechanisms were confirmed using appropriate inhibitors or shRNA. In vitro results were further confirmed by a tumour xenograft study.

**Results:**

CHEPS inhibited the proliferation of NSCLC cells by inducing S‐ and G2/M‐phase arrest and autophagic cell death, but not apoptosis. CHEPS was less toxic to normal human embryonic lung fibroblasts. CHEPS activated the MAPK pathway in NSCLC cells, and p38 and ERK promoted CHEPS‐induced cell death. Further studies showed that p38 and ERK promoted CHEPS‐induced NSCLC cell autophagy and ERK promoted CHEPS‐induced S‐ and G2/M‐phase arrest. ROS were induced by CHEPS. A ROS scavenger attenuated CHEPS‐induced p38 and ERK activation, autophagy and cell death. Finally, CHEPS reduced orthotopic lung tumour growth without organ‐related toxicity. CHEPS also induced ROS, activated p38 and ERK, and triggered autophagy in vivo.

**Conclusions:**

CHEPS induces autophagic cell death and S‐ and G2/M‐phase arrest in NSCLC cells via ROS/p38 and ROS/ERK signalling.

## Graphical abstract

*Cryptococcus heimaeyensis* S20 is found in Antarctica and can produce exopolysaccharides (CHEPS). Hao et al demonstrate that CHEPS induces reactive oxygen species (ROS) generation, which activates p38 and ERK, leading to cell autophagy and death in non‐small cell lung cancer (NSCLC) cells. CHEPS‐activated ERK also induces S‐ and G2/M‐phase arrest.

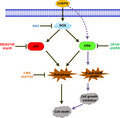

**Abbreviations**
3‐MA: 3‐Methyladenine
AMPK: 5’‐AMP‐activated protein kinase
Baf‐A1: Bafilomycin A1
CCK8: Cell Counting Kit‐8
CHEPS: exopolysaccharide extract from *Cryptococcus heimaeyensis*S20
DAPI: 4′,6‐diamidino‐2‐phenylindole
DCFH‐DA: 2',7'‐Dichlorodihydrofluorescein diacetate
ERK: extracellular signal‐regulated kinase
FBS: foetal bovine serum
H&E: haematoxylin and eosin
JNK: c‐Jun NH2‐terminal kinase
MAPK: mitogen‐activated protein kinase
MDA: malondialdehyde
MEM: Minimum Essential Medium
NAC: N‐acetyl cysteine
NSCLC: non‐small cell lung cancer
PI3K: phosphatidylinositol 3‐kinase
ROS: reactive oxygen species
TEM: transmission electron microscopy

## 1 INTRODUCTION

Lung cancer is the leading cause of cancer‐related death worldwide with increasing morbidity and mortality in recent years.^1^, ^2^ Non‐small cell lung cancer (NSCLC), which includes adenocarcinoma, squamous cell carcinoma and large cell carcinoma, accounts for about 80% of lung cancers and has a low survival rate.^3^ During the past 50 years, substantial progress has been made in the diagnosis and treatment of lung cancer, including screening, diagnostic evaluation, surgery, radiation therapy, traditional chemotherapy, targeted therapy and immunotherapy. Chemotherapy, targeted therapy and immunotherapy are widely applied for treatment of advanced NSCLC.^4^, ^5^ However, the limited efficacy and significant toxicity of these treatments negatively impact the quality of life of patients. Therefore, there is an urgent need to develop innovative anti‐tumour drugs with high efficiency and low toxicity for NSCLC.

Polysaccharides are biopolymers comprised of monosaccharides linked together through glycosidic bonds. Exopolysaccharides (EPS) can be extracted from fungi, bacteria, plants, animals and algae. EPS possess a broad spectrum of biological effects, including anti‐tumour, anti‐viral, immunostimulatory and anti‐oxidative effects.^6^, ^7^, ^8^, ^9^ Increasing evidence indicates that fungal EPS kills cancer cells but has low toxicity to normal cells.^10^, ^11^, ^12^, ^13^ These substances induce apoptosis, autophagy and cell cycle arrest by initiating cell stress responses and altering the expression of signalling molecules in tumour cells.^14^, ^15^, ^16^

Apoptosis, also called type I cell death, is characterized by cell shrinkage, membrane blebbing and chromatin condensation.^17^ Autophagy is a catabolic process, during which parts of the cytosol and specific organelles are engulfed by a double‐membraned structure called the autophagosome. The autophagosome fuses with the lysosome to form an autolysosome, which is where degradation of cellular structures occurs.^18^, ^19^ Autophagy is a double‐edged sword. In normal situations, autophagic activation allows cells to survive during oxidative stress or nutrient deprivation. Under certain conditions, however, excessive autophagy triggers cell death, which is known as autophagic cell death or type II programmed cell death.^20^, ^21^, ^22^ Cell cycle deregulation is a hallmark of tumour cells and the S and G2/M checkpoints are conspicuous targets for anti‐cancer drugs.^21^, ^23^, ^24^ Mitogen‐activated protein kinases (MAPKs) are serine‐threonine kinases that include extracellular signal‐regulated kinase (ERK), p38 and c‐Jun NH2‐terminal kinase (JNK).^25^ Activated MAPKs transmit extracellular signals to regulate cell function.^26^, ^27^ Reactive oxygen species (ROS) are chemically active forms of oxygen that are generally considered to be by‐products of oxygen consumption and cellular metabolism.^28^, ^29^ Some chemotherapeutic drugs induce the accumulation of intracellular ROS, leading to cell apoptosis and autophagy in some tumours^23^, ^24^, ^30^, ^31^

Natural products are a potential source of novel anti‐cancer agents that can be derived from microorganisms. *Cryptococcus heimaeyensis*, which produces EPS to protect itself in cold environments, was first obtained and identified from Icelandic soil in 2002.^32^ However, research on polysaccharides of this genus mainly focuses on *C. neoformans* ^33^ and *C. laurentii*.^34^ *C. neoformans* capsular polysaccharide protects cells from oxidative stress, induces macrophage apoptosis and modulates immune responses.^33^, ^35^, ^36^ *C. heimaeyensis* S20 was isolated from Antarctica; however, the bioactivity of its exopolysaccharide, CHEPS, has not been elucidated. In this study, the biological and molecular mechanisms of the anti‐lung cancer activity of CHEPS are explored in vitro and in vivo.

## 2 MATERIALS AND METHODS

### 2.1 Chemicals and antibodies

CCK‐8 kit and Annexin V‐FITC/PI Apoptosis Detection kit were purchased from Dojindo (Kumamoto, Japan). PI, Glutaraldehyde solution, 2′, 7′‐Dichlorodihydrofluorescein diacetate (DCFH‐DA) and N‐acetyl‐L‐cysteine (NAC) are from Sigma‐Aldrich (St Louis, MO, USA). 3‐Methyladenine (3‐MA), Bafilomycin A1 (Baf‐A1), Dorsomorphin (Compound C), SB202190, U0126 and SP600125 were obtained from Selleck (Houston, Texas, USA).4′,6‐diamidino‐2‐phenylindole (DAPI), Lyso‐Tracker Red and Lipid Peroxidation MDA Assay kit were purchased from Beyotime (Nantong, China).

Antibodies against Cyclin B1 (ET1608‐27), Cyclin A2 (M1511‐5), CDK2 (ET1602‐6), CDK1 (ET1605‐54), ATG5 (ET1611‐38), p38 (ET1602‐26), ERK1/2 (ET1601‐29), phospho‐ERK1/2(Thr202) (ET1610‐13), JNK1/2/3 (ET1601‐28), phospho‐JNK1/2/3(T183 + T183 + T221) (ET1609‐42), AMPK alpha 1 (ET1608‐40), phospho‐AMPK alpha 1 (S496) (ET1612‐72), HSPB1 (ET1701‐70), phospho‐HSPB1(S82) (ET1611‐16) and PARP (ET1608‐56) were purchased from HuaBio (Hangzhou, China). Antibodies against β‐actin (66009‐1‐lg), p53 (10442‐1‐AP), p21 (10355‐1‐AP), p62 (18420‐1‐AP), BAX (50599‐2‐1g) and caspase 3 (19677‐1‐AP) were obtained from Proteintech (Wuhan, China). The antibodies against phospho‐p38 (T180/Y182) (9211S) and LC3B (2775s) were purchased from Cell Signaling Technology (Beverly, MA, USA).

### 2.2 Isolation of the *Cryptococcus heimaeyensis* S20 exopolysaccharide

*C heimaeyensis* S20, which was obtained from the China Center for Type Culture Collection (CCTCC), was incubated in flasks containing YM media and maintained at 180 rpm at 20°C for 5 days. Cultures were then centrifuged. Supernatants were collected and treated with three volumes of 95% ethanol and maintained at 4°C overnight. Most of the supernatant solution was decanted, and the remaining liquid was centrifuged to remove the supernatant. The precipitate was freeze‐dried to obtain the crude exopolysaccharide powder. The powder was dissolved in distilled water and deproteinated using the Sevag method. The aqueous phase was added to a 3‐kDa ultrafiltration tube and centrifuged to obtain an exopolysaccharide solution from which small molecules had been removed, and this was then lyophilized. The powder was the *C heimaeyensis* S20 exopolysaccharide, which we refer to as CHEPS.

### 2.3 Cell lines and cell culture

Human embryonic lung fibroblast cell lines WI‐38 and MRC‐5, human lung adenocarcinoma cell lines A549 and NCI‐H1299, and human lung squamous cell line SK‐MES‐1 were provided by CCTCC and cultured in Minimum Essential Medium (MEM; Gibco, CA, USA) supplemented with 10% heat‐inactivated foetal bovine serum (FBS; Sijiqing, Hangzhou, China) at 37°C with 5% CO2.

### 2.4 Cell viability assay

Cell suspensions (100 μl; 5 × 10^4^ cells/ml) were seeded in 96‐well plates and allowed to grow for 24 h before CHEPS treatment at various concentrations (500, 250, 125 and 62.5 μg/ml), and 5‐Fluorouracil (100 μg/ml) was used as a positive control. After 0, 24, 48 and 72 h, 10 μl CCK8 solution was added to each well. Samples were incubated at 37°C for 1 h before absorbance was measured at a wavelength of 450 nm.

### 2.5 Colony formation assay

Cells were seeded in 6‐well plates at a density of 1,000 cells per well for A549, NCI‐H1299, and SK‐MES‐1, and 10,000 cells for WI‐38 and MRC‐5. After 48 h, various concentrations of CHEPS were added, and cells were allowed to grow for 8‐10 days until visible colonies formed. Colonies were fixed with methyl alcohol and stained with crystal violet. Finally, the crystal violet was dissolved in 70% ethanol, and absorbance was measured at 570‐nm wavelength to calculate the colony formation rate.

### 2.6 Cell cycle analysis

Cells were seeded in 6‐well plates with a density of 3 × 10^5^ cells/ml and allowed to grow for 24 h before treatment with various concentrations of CHEPS (500, 250, 125 μg/ml) for 48 h. Cells were harvested and fixed with cold 70% ethanol at −20°C overnight. Cells were then suspended in staining buffer (50 µg/ml PI, 20 µg/ml RNase A in PBS) for 20 min at room temperature (RT). PI‐stained cells were analysed by flow cytometry (CyAn ADP, Beckman Coulter, USA).

### 2.7 Cell apoptosis analysis

Annexin V‐FITC/PI Apoptosis Detection kit was used to detect phosphatidylserine evagination. All kit reagents were used according to the manufacturer's instructions. Briefly, cells were harvested and stained by Annexin V‐FITC/PI. Then, cells were analysed by flow cytometry.

### 2.8 Autophagy detection

For the EGFP‐LC3 puncta assay, pEGFP‐LC3B was constructed and transfected into cells for 24 h before CHEPS treatment. Cells were fixed with 4% paraformaldehyde for 20 min at room temperature. DAPI was used to stain the nucleus for 5 min. Cells were then observed under a Leica SP8 confocal microscope (Leica, Germany) and images were analysed with LAS AF Lite software. Autophagic cells, which were defined as cells with five or more EGFP‐LC3B green dots, were counted.^37^ For Lyso‐Tracker Red staining, cells were treated with CHEPS and incubated with 50 nM Lyso‐Tracker Red in the dark for 40 min at 37°C. Samples were analysed using a Leica SP8 confocal microscope.

### 2.9 Measurement of ROS

Cells were treated with CHEPS with or without 5 mM NAC. Cells were then incubated with DCFH‐DA at a final concentration of 10 μM in MEM without FBS for 1 h at 37°C and washed three times with MEM. The level of ROS was determined by fluorescence microscopy and flow cytometry.

### 2.10 Transmission electron microscopy (TEM)

Cell or tumour samples were fixed in 2.5% glutaraldehyde and washed three times in PBS the next day. Samples were then fixed with 1% osmium acid at 4°C for 2‐3 h followed by washing in PBS and dehydration through an alcohol gradient. Successive permeation was then performed using acetone:epoxy resin (2:1), acetone:epoxy resin (1:1) and epoxy resin. Samples were embedded into epoxide resin, sliced with an EM UC7 ultramicrotome (Leica, Germany) and observed with a Tecnai G2 20 TWIN transmission electron microscope (FEI, USA).

### 2.11 RNA extraction, qRT‑PCR and RNAi

Total RNA was extracted from A549 and WI‐38 cells using TRIzol (Invitrogen, USA) according to the manufacturer's instructions. Reverse transcription was performed using murine MLV reverse transcriptase (Promega, USA). Quantitative PCR was performed with a CFX96 real‐time PCR detection system (Bio‐Rad, USA). The qPCR primers used are listed in Table S1.

Oligonucleotides were cloned into pSUPER‐retro‐puro plasmid for RNA interference. The ATG5 shRNA target sequence was GGATGCAATTGAAGCTCAT. The p38 shRNA target sequence was GCCTGACCTATGATGAAGT. The ERK shRNA target sequence was GAAGACCTGAATTGTATAA. The AMPK shRNA target sequence was GGTTGTAAACCCATATTAT. The negative control sequence was TTCTCCGAACGTGTCACGT. The shATG5, shp38, shERK and shAMPK retroviral A549 and NCI‐H1299 stable cell lines were established as previously described.^38^

### 2.12 Western blot analysis

Cells treated with CHEPS were collected and lysed in 1% SDS buffer. Cell lysates were separated by SDS‐PAGE and transferred to a PVDF membrane (Millipore, USA). After blocking with 5% non‐fat milk for 2 h, the membranes were incubated with primary antibody at 4°C overnight. Membranes were then washed with TBST buffer and incubated with HRP‐conjugated secondary antibody for 1 h at RT. Specific antibody binding was detected by ECL Chemiluminescence (Millipore, USA).

### 2.13 In vivo tumour xenograft study

Female BALB/c‐nu mice (4 weeks old) were purchased from Beijing Vital River Laboratory Animal Technology Co., Ltd (Beijing, China). The protocol was approved by the Committee on the Ethics of Animal Experiments of Wuhan University (Permit Number 18050C). Mice were housed in a specific pathogen‐free (SFP) environment with a 12 h day/night cycle at RT (22 ± 2°C). A549 cells (5 × 10^6^) suspended in 0.2 ml MEM were inoculated subcutaneously in the right axilla of each mouse. After 3 days, mice were randomly distributed into two groups, a control group and a CHEPS group, with 10 mice per group. The control group received intraperitoneal injection of MEM every other day, and the CHEPS group received CHEPS (100 mg/kg, diluted in MEM). Tumour volume and body weight were measured every other day. Tumour volumes were calculated by the following formula: 0.5 × large diameter × (small diameter).^2^ After administering the drug 20 times, mice were sacrificed by cervical dislocation, and tumours were removed and weighted. A section of tumour tissue was fixed for immunohistochemical and TEM examination. Another section was stored at −80°C for use in the MDA assay as described in the Lipid Peroxidation MDA Assay kit. Tissue samples from the liver, spleen, lung and kidney were also stripped and fixed.

### 2.14 Histopathology and immunohistochemistry

Formalin‐fixed tissue samples were embedded in paraffin, and 4‐μm sections were cut. Primary tumours, liver, spleen, lung and kidney sections were stained with H&E for histological examination and morphometric analysis. For immunohistochemical staining, tumour slides were deparaffinized in xylene and rehydrated with graded alcohol. Tissues were then incubated in 3% hydrogen peroxide to block endogenous peroxidase activity. Antigen retrieval was performed by boiling slides in 10 mM sodium citrate (pH 6.0) for 30 min. Slides were then blocked in 10% normal goat serum for 15 min, followed by incubation with antibodies against LC3B, p62, P‐ERK and P‐p38 at 4°C overnight in a moist chamber. The next day, the slides were washed in PBS and incubated with the secondary antibody for 1 h at RT. Immunoreactivity was detected using a DAB kit.

### 2.15 Statistical analysis

Quantitative data are presented as mean ± SD. Student's t test and one‐way ANOVA were used to calculate the differences between two groups. A value of *P* < .05 was considered statistically significant.

## 3 RESULTS

### 3.1 CHEPS inhibits the proliferation of NSCLC cells and is less cytotoxic to normal human embryonic lung cells

**Figure 1:**
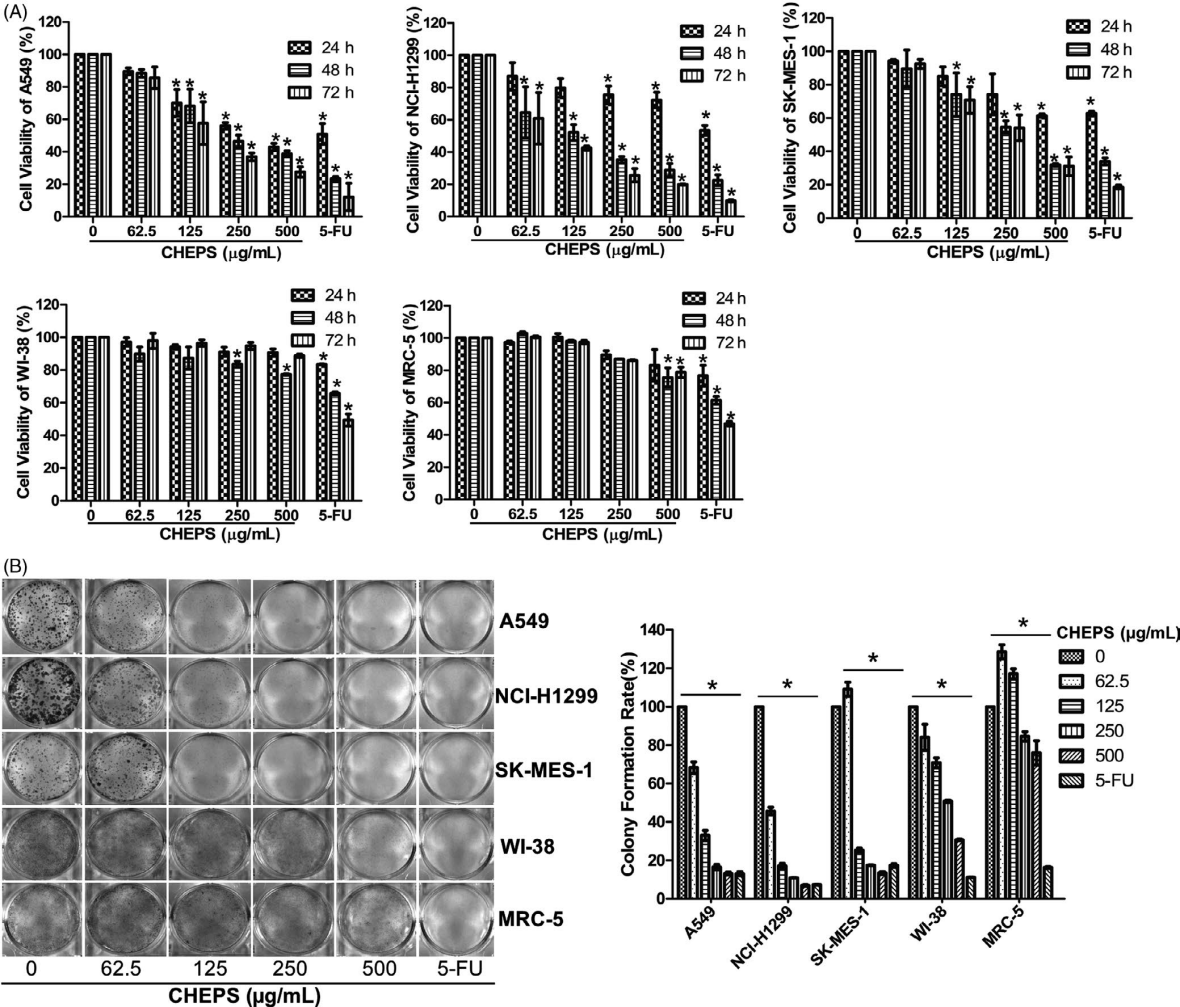
CHEPS inhibits the proliferation and colony‐forming ability of NSCLC cells with little cytotoxicity in normal human embryonic lung cells. A, CCK8 assay was used to evaluate cell proliferation. Three NSCLC cell lines (A549, NCI‐H1299, SK‐MES‐1) and two normal human embryonic lung cell lines (WI‐38, MRC‐5) were treated with various concentrations of CHEPS for 24 to 72 h. B, Colony formation assay was performed in A549, NCI‐H1299, SK‐MES‐1, WI‐38 and MRC‐5 cells with or without CHEPS treatment for 8‐12 days. 5‐FU (100μg/ml) was used as a positive control. Data represented the mean ± SD from three independent experiments. ^*^ *P* < .05, significantly different compared with CHEPS (0 μg/ml)

CHEPS reduced NSCLC cell viability in a dose‐dependent and time‐dependent manner (Figure 1A). The percentage of viable cells was 27.62% in A549 cells, 20.03% in NCI‐H1299 cells and 36.11% in SK‐MES‐1 cells after incubation with 500 μg/ml CHEPS for 72 h. By contrast, the percentage of viable normal human embryonic lung fibroblasts WI‐38 and MRC‐5 was 88.76% and 78.75%, respectively. Almost no colonies were formed after 125 μg/ml CHEPS treatment in A549, NCI‐H1299 and SK‐MES‐1 cells. Interestingly, no obvious changes in colony formation were observed in WI‐38 and MRC‐5 cells after 125 μg/ml CHEPS treatment (Figure 1B). These results suggest that CHEPS inhibits the proliferation and colony‐forming ability of NSCLC cells with little cytotoxicity in normal human embryonic lung cells.

### 3.2 CHEPS does not induce apoptosis but triggers autophagy

**Figure 2:**
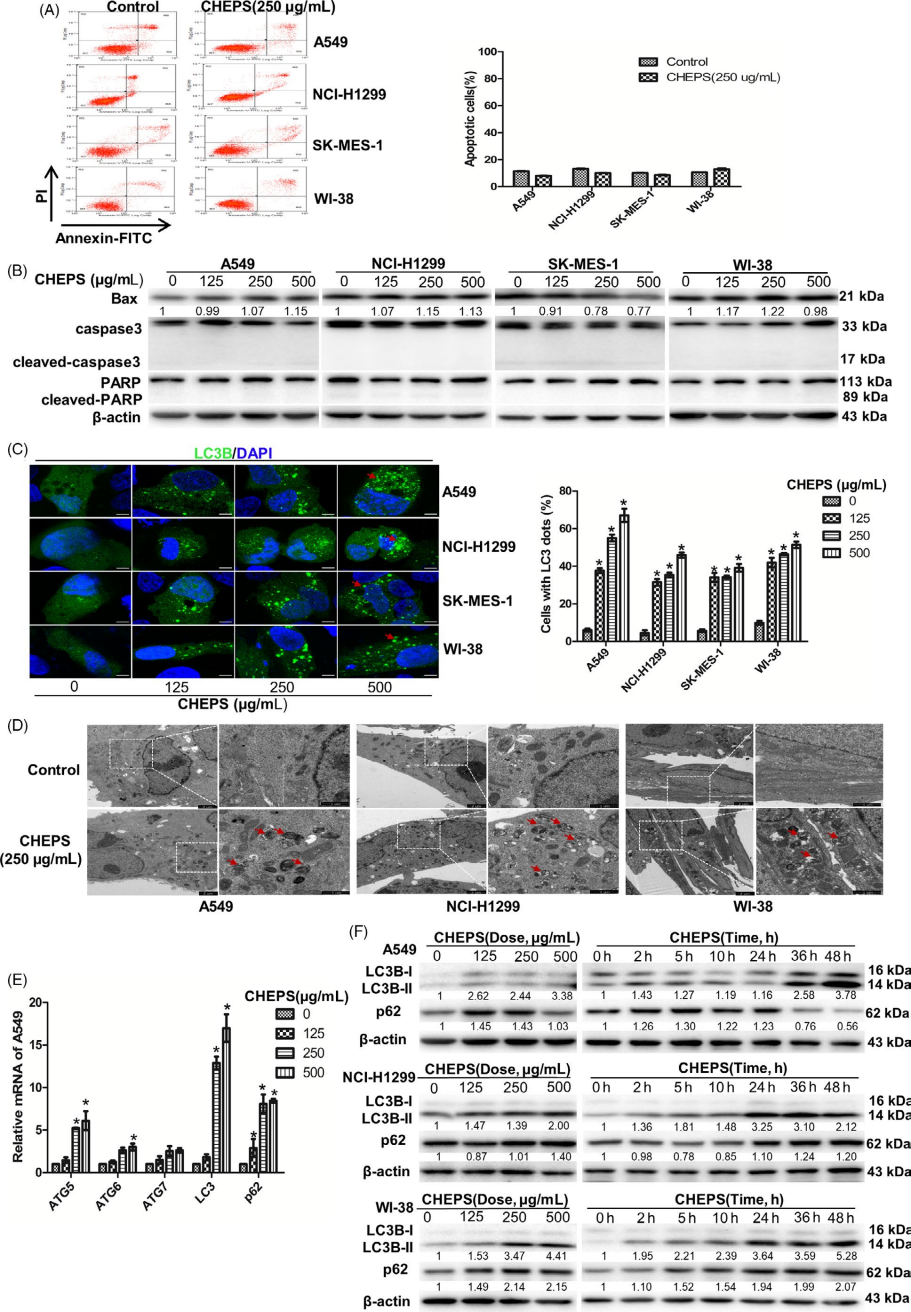
CHEPS does not induce cell apoptosis but triggers cell autophagy. A, A549, NCI‐H1299, SK‐MES‐1 and WI‐38 cells treated with CHEPS (250 µg/ml) for 48 h were stained with Annexin V‐FITC/PI and analysed by flow cytometry. The chart illustrates apoptosis from three separate experiments. B, A549, NCI‐H1299, SK‐MES‐1 and WI‐38 cells were treated with CHEPS (0, 125, 250, 500 µg/ml) for 48 h. The expression levels of apoptosis‐related proteins were analysed by Western blot. C, A549, NCI‐H1299, SK‐MES‐1 and WI‐38 cells were pre‐transfected with the pEGFP‐LC3B plasmid for 24 h followed by treatment with or without CHEPS (0, 125, 250, 500 µg/ml) for 5 h and observation under a confocal microscope. Images show the cellular localization patterns of the EGFP‐LC3B fusion protein. At least 150 EGFP‐LC3B‐transfected cells were counted in each experiment. Arrows indicate autophagosomes. Bar: 10 µm. *P < .05, significantly different compared with CHEPS (0 μg/ml). D, Transmission electron microscopy was utilized to observe autophagosome formation. Arrows indicate autophagosomes and autolysosomes containing intact and degraded cellular debris. Bar: 1 and 2 µm. E, A549 cells were treated with CHEPS (0, 125, 250, 500 µg/ml) for 48 h. The mRNA levels of autophagy‐related genes were analysed by qRT‐PCR. *P < .05 versus CHEPS (0 μg/ml). F, Cells were treated with various concentrations of CHEPS for 24 h or incubated with CHEPS (250 µg/ml) for different time points. The expression levels of LC3B and p62 were determined by Western blot

Apoptosis contributes to cell death; however, CHEPS did not induce apoptosis in NSCLC or WI‐38 cells based on flow cytometry analysis of Annexin V‐FITC/PI staining (Figure 2A). Western blot analyses confirmed that CHEPS did not upregulate the expression of Bax or induce caspase‐3 and PARP cleavage (Figure 2B). These data suggest that CHEPS‐induced cell death does not occur via apoptosis.

Accumulating evidence suggests that the effects of anti‐cancer therapies also involve autophagy. During autophagy, LC3 is converted from LC3‐I to LC3‐II and is positioned on the membrane of autophagosomes.^39^ A EGFP‐LC3B fusion protein was used to monitor the location of LC3 particles to determine if CHEPS induces autophagy. Large numbers of LC3B puncta appeared after CHEPS treatment in a dose‐dependent manner in NSCLC and WI‐38 cells (Figure 2C). Next, the acidotropic dye probe Lyso‐Tracker Red was used to label cellular acidic compartments. After exposure to CHEPS for 5 h, the fluorescence intensity increased in NSCLC and WI‐38 cells, indicating the production of autolysosomes (Figure S1A). TEM examination showed the presence of a large number of autophagosomes and autolysosomes in the cytoplasm of NSCLC and WI‐38 cells after treatment with CHEPS for 24 h (Figure 2D). Further, the mRNA levels of autophagy‐related genes, including ATG5, ATG6, ATG7, LC3 and p62, were upregulated in A549 cells after CHEPS treatment (Figure 2E). In addition, CHEPS increased LC3B‐II protein levels and regulated the expression of p62 in a dose‐dependent and time‐dependent manner in NSCLC and WI‐38 cells (Figure 2F). These results demonstrate that CHEPS triggers cell autophagy rather than apoptosis.

### 3.3 CHEPS enhances autophagic flux and induces autophagic cell death in NSCLC cells

**Figure 3:**
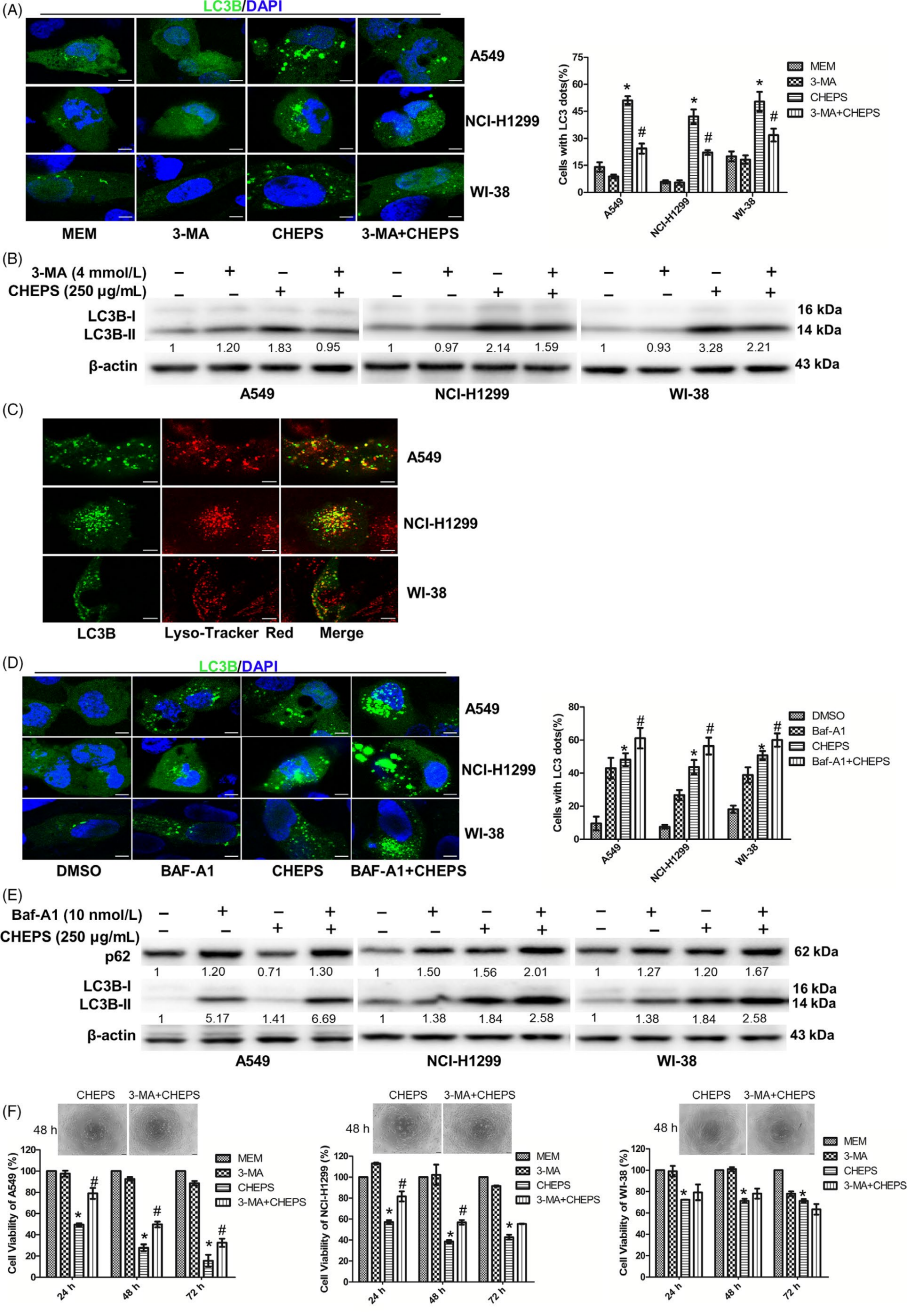
CHEPS enhances autophagic flux and induces autophagic cell death in non‐small cell lung cancer cells. A, After pre‐incubation with 4 mM 3‐MA for 2 h, A549, NCI‐H1299 and WI‐38 cells pre‐transfected with the pEGFP‐LC3B plasmid for 24 h were treated with 250 µg/ml CHEPS with or without 3‐MA for 5 h followed by observation under a confocal microscope. At least 150 EGFP‐LC3B‐transfected cells were counted in each experiment. Bar: 10 µm. *P < .05 versus MEM, # *P* < .05 versus CHEPS treatment. B, After pre‐incubation with 4 mM 3‐MA for 2 h, A549, NCI‐H1299 and WI‐38 cells were treated with 250 µg/ml CHEPS with or without 3‐MA for 5 h. Cell lysates were examined for LC3B by Western blotting. C, A549, NCI‐H1299 and WI‐38 cells were pre‐transfected with the pEGFP‐LC3B plasmid for 24 h and then treated with CHEPS (250 µg/ml) for 5 h followed by Lyso‐Tracker Red staining and observation under a confocal microscope. Images show the cellular colocalization patterns of the EGFP‐LC3B fusion protein and autolysosomes. Bar: 10 µm. D, After pre‐incubation with 10 nM Baf‐A1 for 1 h, A549, NCI‐H1299 and WI‐38 cells pre‐transfected with the EGFP‐LC3B plasmid for 24 h were treated with 250 µg/ml CHEPS with or without Baf‐A1 for 5 h followed by observation under a confocal microscope. At least 150 EGFP‐LC3B‐transfected cells were counted in each experiment. Bar: 10 µm. *P < .05 versus DMSO, # *P* < .05 versus CHEPS treatment. E, After pre‐incubation with 10 nM Baf‐A1 for 1 h, A549, NCI‐H1299 and WI‐38 cells were treated with 250 µg/ml CHEPS with or without Baf‐A1 for 24 h. Cell lysates were examined for p62 and LC3B expression by Western blot analysis. F, 3‐MA (4 mM) was added to A549, NCI‐H1299 and WI‐38 cells 2 h before CHEPS (250 µg/ml) treatment. After 24 h, 48 h and 72 h time points, cell viability was determined by CCK8 assay and cell morphology was observed under a microscope. Data represent the mean ± SD from three independent experiments. Bar: 100 µm. *P < .05 versus MEM, # *P* < .05 versus CHEPS treatment

To further confirm that CHEPS induced autophagy, we treated cells with 3‐methyladenine (3‐MA), a specific inhibitor of autophagy initiation. 3‐MA significantly blocked the formation of EGFP‐LC3B fluorescent puncta and repressed the expression of LC3B‐II (Figure 3A, B). To test whether autophagosome production was due to increased induction of autophagy or blockade of autophagic flux, we stained EGFP‐LC3B‐transfected A549, NCI‐H1299 and WI‐38 cells with Lyso‐Tracker Red. We found that the green EGFP‐LC3B puncta were partially colocalized with the red Lyso‐Tracker puncta, indicating fusion of autophagosomes with lysosomes (Figure 3C). Next, we treated NSCLC and WI‐38 cells with Bafilomycin A1 (Baf‐A1), a lysosome inhibitor that inhibits autophagy degradation. EGFP‐LC3B fluorescent puncta and expression of p62 and LC3B‐II further increased after CHEPS plus Baf‐A1 treatment compared with either treatment alone (Figure 3D, E). These data demonstrate that CHEPS induces complete autophagic flux.

Autophagy can promote cell survival or contribute to cell death.^20^, ^22^ To clarify the role of autophagy on CHEPS‐induced cell death, we used the autophagy inhibitor 3‐MA, and assessed cell viability. 3‐MA remarkably diminished CHEPS‐induced cell death in A549 and NCI‐H1299 cells. However, it did not significantly reverse CHEPS‐induced cell viability reduction‐ in WI‐38 cells (Figure 3F). To further confirm that CHEPS induced autophagic cell death in NSCLC cells, we established shATG5‐stable cell lines of A549 and NCI‐H1299. CHEPS did not induce the formation of EGFP‐LC3B puncta or increase LC3B‐II expression in shATG5‐stable A549 and NCI‐H1299 cells (Figure S1B, C). Further, shATG5 moderately rescued CHEPS‐induced cell death by ~ 12% in A549 cells and ~ 8% in NCI‐H1299 cells (Figure S1D). These results suggest that CHEPS induces autophagic cell death in NSCLC cells. Although CHEPS induced autophagy in WI‐38 cells, it did not induce autophagic cell death.

### 3.4 CHEPS induces MAPK activation, and p38 and ERK mediate CHEPS‐induced cell death

**Figure 4:**
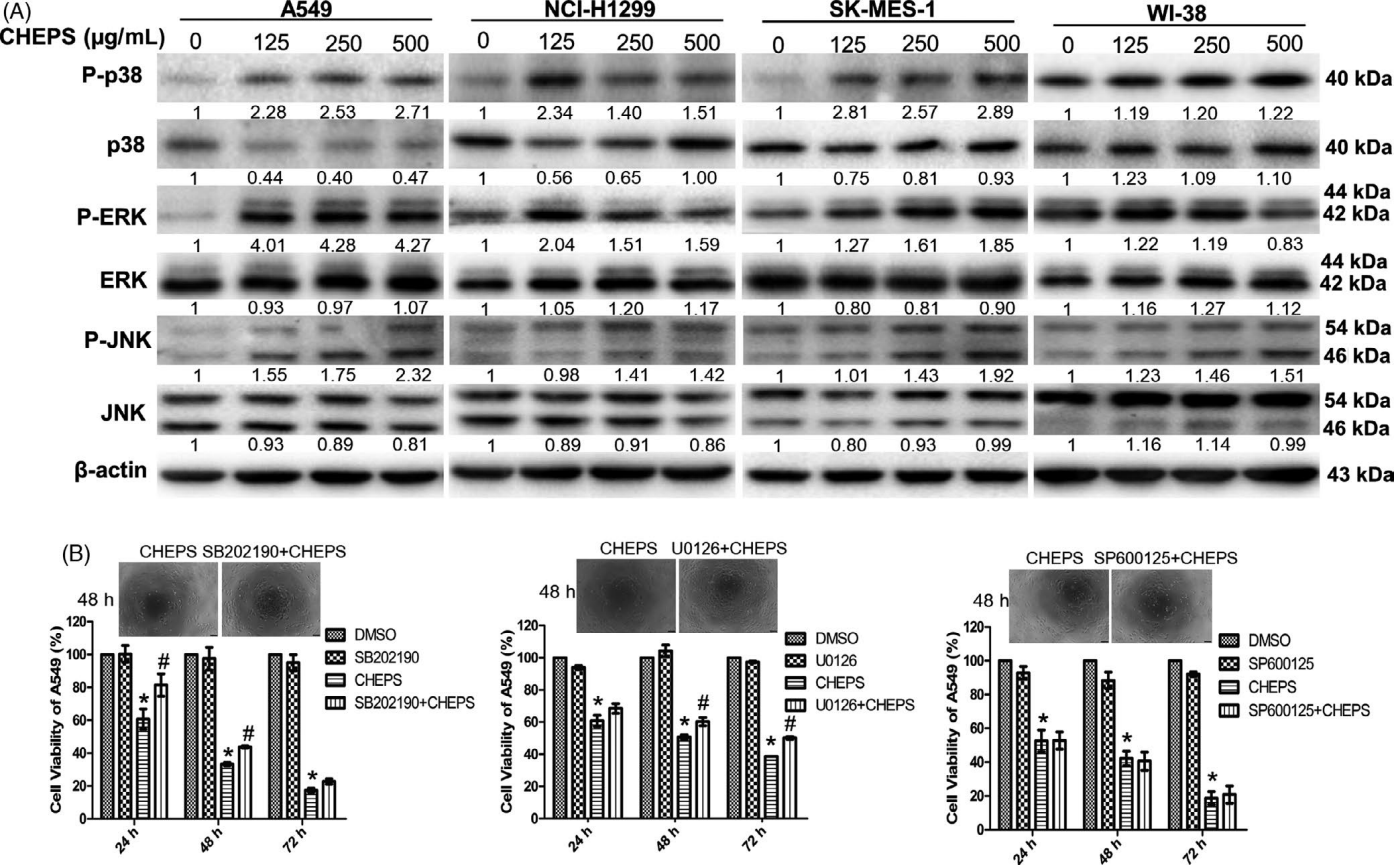
CHEPS induces MAPK pathway activation, of which p38 and ERK contribute to CHEPS‐induced cell death. A, A549, NCI‐H1299, SK‐MES‐1 and WI‐38 cells were treated with CHEPS (0, 125, 250, 500 µg/ml) for 48 h. Cell lysates were examined for MAPK‐related proteins by Western blot. B, SB202190 (2 µM, p38 inhibitor), U0126 (2 µM, ERK inhibitor) and SP600125 (5 µM, JNK inhibitor) were added to A549 cells 2 h before CHEPS (250 µg/ml) treatment. After 24 h, 48 h, and 72 h, cell viability was determined by CCK8 assay and cell morphology was observed under a microscope. Bar: 100 µm. Data represent the mean ± SD from three independent experiments. *P < .05 versus DMSO, # *P* < .05 versus CHEPS treatment

We examined whether the MAPK pathway was involved CHEPS‐induced cell death. We found that CHEPS induced phosphorylation of p38, ERK and JNK in a dose‐dependent and time‐dependent manner in NSCLC cells. Interestingly, CHEPS did not significantly induce MAPK pathway activation in WI‐38 cells (Figure 4A, Figure S2A). To determine the individual contributions of activated MAPK signalling molecules to CHEPS‐induced cell death, we used SB202190, an ATP‐competitive p38 inhibitor, U0126, a MEK1/2 inhibitor that inhibits ERK2 phosphorylation, and SP600125, a JNK inhibitor that inhibits c‐Jun phosphorylation. SB202190 and U0126 rescued CHEPS‐induced cell death in A549 cells. However, SP600125 had no effect on cell death (Figure 4B). SB202190 and U0126 also rescued CHEPS‐induced cell death in NCI‐H1299 cells (Figure S2B). CHEPS exhibited weaker inhibitory effects in A549 and NCI‐H1299‐ shp38‐ or shERK‐stable cell lines compared with NC cells (Figure S2C). The AMPK pathway has been reported to regulate autophagy.^40^ CHEPS was seen to activate AMPK signalling (Figure S3A). However, activated AMPK had no effect on CHEPS‐induced cell death and cell autophagy in NSCLC cells (Figure S3B–E). These data suggest that CHEPS activates p38, ERK, and JNK in NSCLC cells, and that p38 and ERK contribute to CHEPS‐induced cell death. Although CHEPS activates JNK and AMPK, they do not appear to be involved in CHEPS‐induced cell death.

### 3.5 p38 and ERK regulate CHEPS‐induced cell autophagy

**Figure 5:**
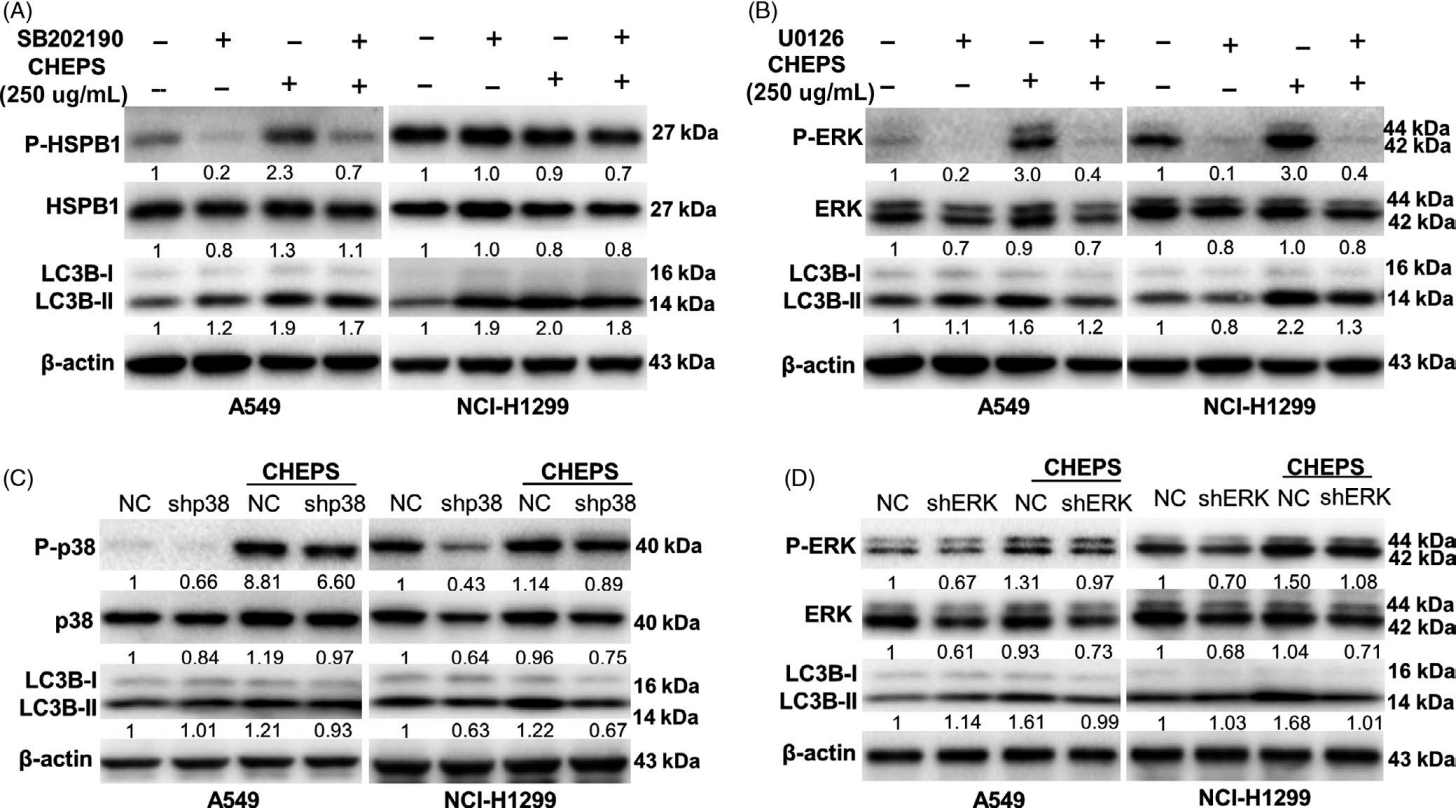
p38 and ERK regulate CHEPS‐induced cell autophagy. A, A549 and NCI‐H1299 cells were pre‐incubated with SB202190 (2 µM) for 2 h followed by CHEPS (250 µg/ml) treatment for 24 h. Cell lysates were collected, and expression of LC3B protein was analysed by Western blot. SB202190 inhibits p38 catalytic activity by binding to the ATP binding pocket of p38, but it does not disturb the phosphorylation of p38 by upstream kinases, we therefore examined phosphorylation of HSPB1, which is downstream of p38, to determine if p38 was inactivated. B, A549 and NCI‐H1299 cells were pre‐incubated with U0126 (2 µM) for 2 h followed by CHEPS (250 µg/ml) treatment for 24 h. Cell lysates were collected, and expression of LC3B protein was analysed by Western blot. C, CHEPS (250 µg/ml) was added to A549 and NCI‐H1299 pre‐transfected with pSUPER‐shp38 for 24 h. Cell lysates were collected, and expression of LC3B protein was analysed by Western blot. D, CHEPS (250 µg/ml) was added to A549 and NCI‐H1299 pre‐transfected with pSUPER‐shERK for 24 h. Cell lysates were collected, and expression of LC3B protein was analysed by Western blot

Next, we examined whether p38‐ and ERK‐regulated CHEPS‐induced cell death involved cell autophagy. Although SB202190 alone promoted autophagy,^41^ it did not enhance CHEPS‐mediated autophagy in A549 and NCI‐H1299 cells, suggesting that SB202190 weakened CHEPS‐induced autophagy (Figure 5A). U0126 largely abrogated the increased expression of LC3B‐II induced by CHEPS in A549 and NCI‐H1299 cells (Figure 5B). Similarly, the increased expression levels of LC3B‐II induced by CHEPS in A549 and NCI‐H1299‐shp38 or ‐shERK cells were partly abrogated compared with NC cells (Figure 5C, D). These results demonstrate that p38 and ERK promote NSCLC cell death by regulating CHEPS‐induced cell autophagy.

### 3.6 CHEPS induces ROS generation, which activates p38 and ERK and induces cell autophagy and death

**Figure 6:**
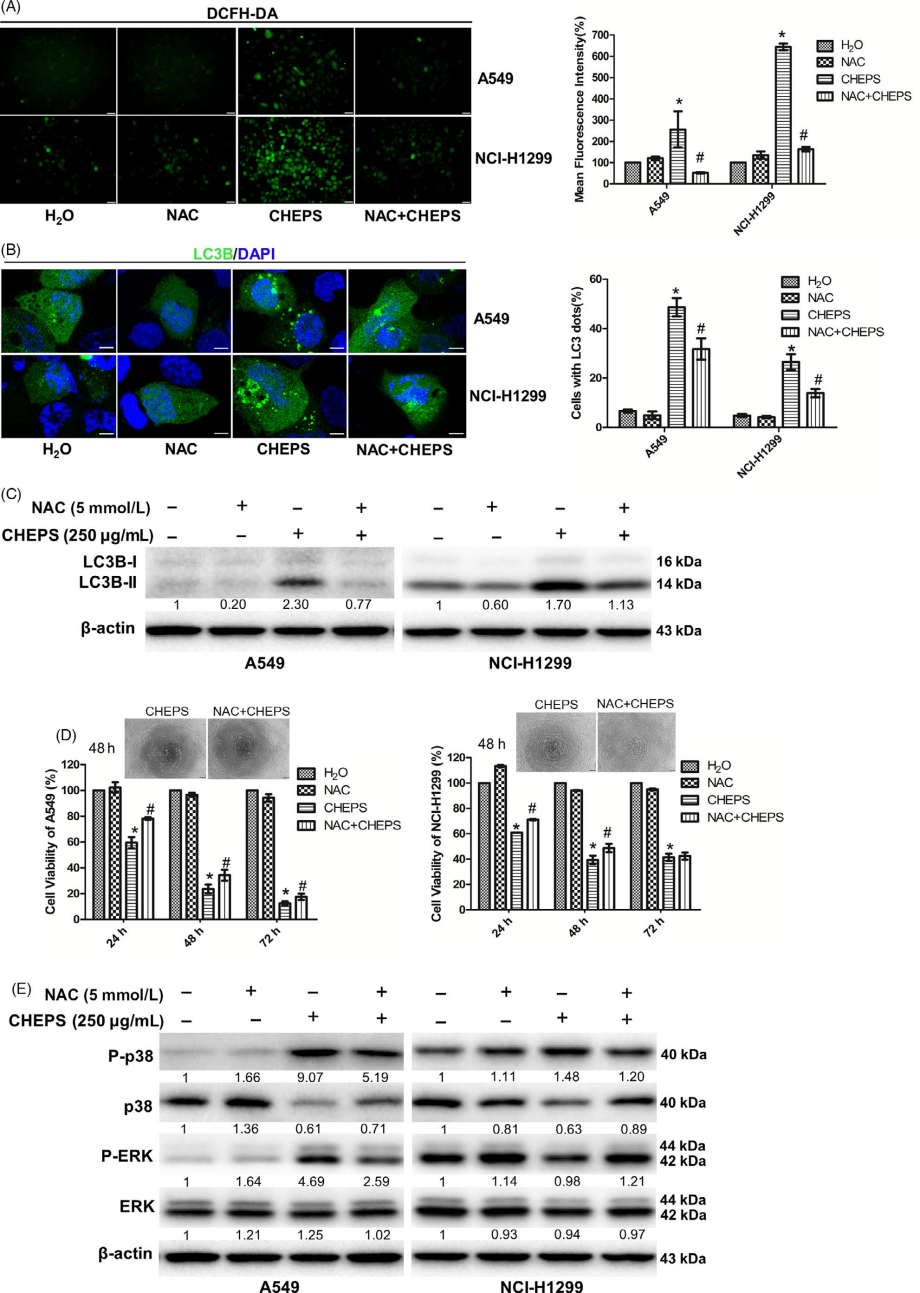
CHEPS induces ROS generation, which activates p38 and ERK signalling and induces cell autophagy and death. A, After incubation with NAC (5 mM) for 2 h, A549 and NCI‐H1299 cells were treated with 250 µg/ml CHEPS with or without NAC for another 24 h. Cells were stained with DCFH‐DA and observed by fluorescence microscopy. The fluorescence intensity was analysed using ImageJ software. Bar: 50 µm. *P < .05 versus H_2_O, # *P* < .05 versus CHEPS treatment. B, After incubation with NAC (5 mM) for 2 h, A549 and NCI‐H1299 cells pre‐transfected with pEGFP‐LC3B for 24 h were treated with 250 µg/ml CHEPS with or without NAC for another 5 h followed by observation under a confocal microscope. At least 150 EGFP‐LC3B‐transfected cells were counted in each experiment. Bar: 10 µm. *P < .05 versus H_2_O, # *P* < .05 versus CHEPS treatment. C, After incubation with NAC (5 mM) for 2 h, A549 and NCI‐H1299 cells were treated with 250 µg/ml CHEPS with or without NAC for another 24 h. Cell lysates were collected, and expression level of LC3B protein was analysed by Western blotting. D, After incubation with NAC (5 mM) for 2 h, A549 and NCI‐H1299 cells were treated with 250 µg/ml CHEPS with or without NAC for another 24 h to 72 h. Cell viability was determined by CCK8 assay and cell morphology was observed under a microscope. Data represent the mean ± SD from three independent experiments. Bar: 100 µm. *P < .05 versus H_2_O, # *P* < .05 versus CHEPS treatment. E, After incubation with NAC (5 mM) for 2 h, A549 and NCI‐H1299 cells were treated with 250 µg/ml CHEPS with or without NAC for another 24 h. Cell lysates were collected, and expression levels of total and phosphorylated p38 and ERK were analysed by Western blotting

.We next investigated whether CHEPS‐induced NSCLC cell death is associated with ROS. NSCLC and WI‐38 cells were treated with CHEPS for 24 h and then stained with DCFH‐DA, a ROS‐specific fluorescent dye. Fluorescence intensity increased in NSCLC but not in WI‐38 cells according to fluorescence microscopy (Figure S4A) and flow cytometry analysis (Figure S4B). This increase was completely blocked by pretreatment with the ROS scavenger NAC (Figure 6A). Pretreatment with NAC significantly abolished the formation of EGFP‐LC3B fluorescent puncta (Figure 6B) and expression of LC3B‐II in A549 and NCI‐H1299 cells (Figure 6C), suggesting that CHEPS‐triggered autophagy was induced by elevated levels of ROS. Moreover, CHEPS‐induced autophagic cell death was via ROS, with ROS elimination by NAC partly restoring cell viability (Figure 6D). ROS have been reported to regulate MAPK signalling,^42^, ^43^ and we suggest that this regulation may also apply to the activation of p38 and ERK induced by CHEPS in NSCLC cells. As expected, NAC partly reduced upregulation of phospho‐p38 and phospho‐ERK induced by CHEPS in A549 cells and the upregulation of phospho‐p38 in NCI‐H1299 cells (Figure 6E). These data suggest that CHEPS induces ROS generation in NSCLC cells, which activates p38 and ERK signalling and induces cell autophagy and death.

### 3.7 CHEPS induces S‐ and G2/M‐phase arrest through ERK

Cell cycle arrest can lead to cell proliferation inhibition. In our study, CHEPS significantly increased the percentage of NSCLC cells in S‐ and G2/M‐phases. Cell cycle arrest occurred but was less pronounced in WI‐38 cells compared with NSCLC cells (Figure S5A). The mRNA and protein levels of p53 and p21 were upregulated, and Cyclin B1, CDK1, Cyclin A2 and CDK2 were significantly downregulated in NSCLC cells following CHEPS treatment for 48 h. These changes were less prominent in WI‐38 cells with the exception of p21 (Figure S5B‐D). These data indicate that CHEPS triggers S‐ and G2/M‐phase arrest by altering the expression of cell cycle regulatory proteins.

Flow cytometry analyses showed that U0126 partly restored CHEPS‐induced S‐ and G2/M‐phase arrest in A549 cells, whereas SB202190 did not (Figure S6A). Accordingly, U0126 partly rescued CHEPS‐induced upregulation of p21 and downregulation of CDK2 in A549 cells (Figure S6B). These data suggest that ERK also promotes NSCLC cell death by regulating CHEPS‐induced cell cycle arrest. Nonetheless, NAC did not rescue S‐ and G2/M‐phase arrest induced by CHEPS (Figure S6C, D), indicating that ERK‐promoted cell cycle arrest is not via ROS.

### 3.8 CHEPS induces NSCLC cell autophagy and inhibits tumour growth in vivo

**Figure 7:**
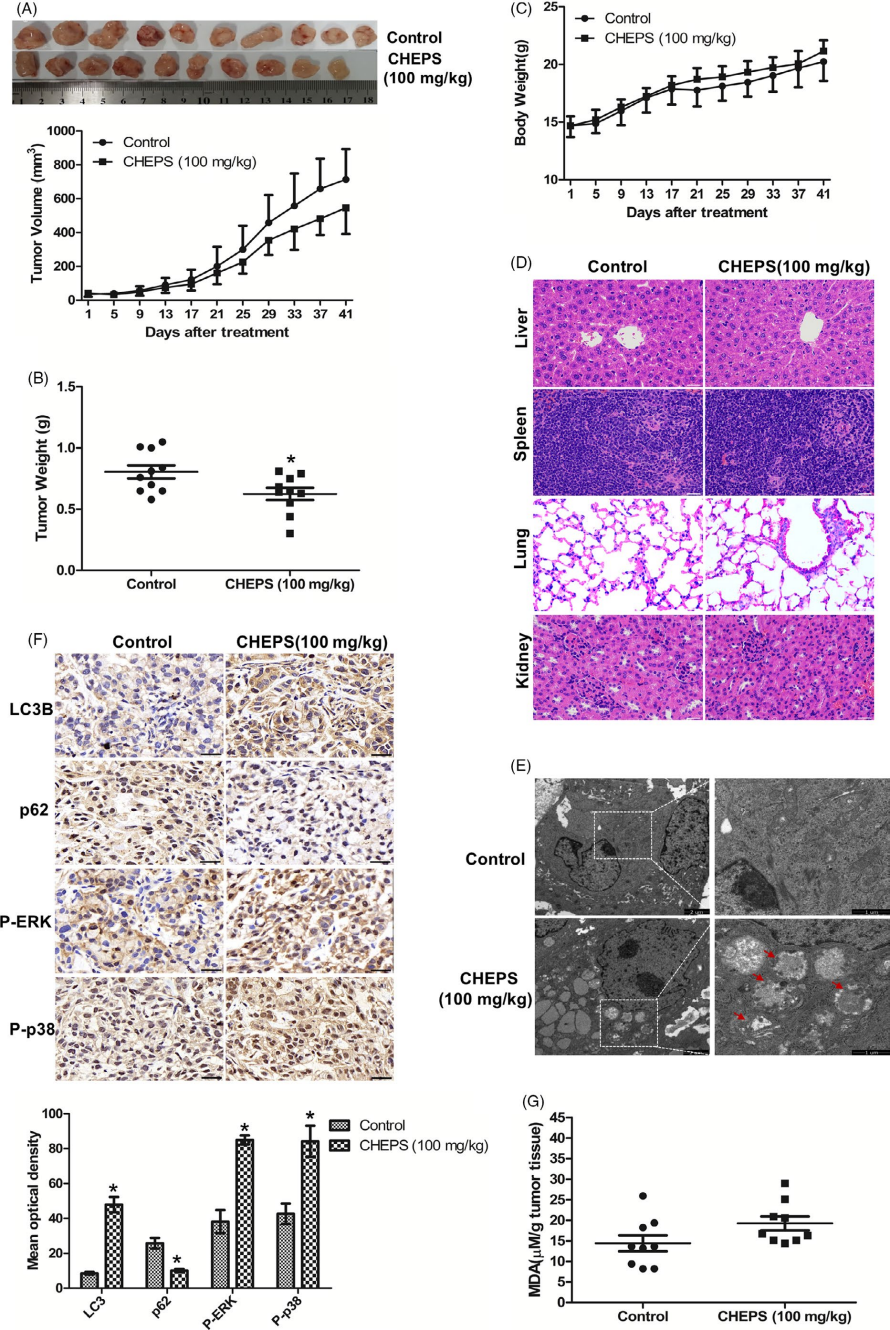
CHEPS induces NSCLC cell autophagy and inhibits tumour growth in vivo. A549 cells (5 × 10^6^) were inoculated subcutaneously in the right axilla of 20 female BALB/c‐nu mice. After 3 days, mice were randomly distributed into two groups, a control group and CHEPS group, with 10 mice in each group. The control group received intraperitoneal injection of MEM every other day, and the CHEPS group was injected with CHEPS (100 mg/kg, diluted in MEM). After the drug was administered 20 times, mice were sacrificed by cervical dislocation. Tumours were removed, weighed and fixed for immunohistochemical experiments and TEM examination. Tissue samples from the liver, spleen, lung and kidney were also stripped and fixed for H&E staining. A, Tumour size was measured. B, Tumours were removed and weighed after mice were sacrificed. C, Body weights were measured. D, The histology of liver, spleen, lung and kidney were evaluated by H&E staining. Bar: 50 µm. E, The tumours were fixed and used for TEM examination. Bar: 1 and 2 µm. F, The expression of LC3B, p62, P‐ERK and P‐p38 were examined by immunohistochemistry. Bar: 50 µm. Mean optical density of LC3B, p62, P‐ERK and P‐p38 was quantified by Image Pro‐Plus. *P < .05, significantly different compared with control. G, MDA levels were examined in tumour tissue

To further assess the anti‐tumour effects of CHEPS, an in vivo orthotopic xenograft model of NSCLC cells was established by subcutaneous inoculation of A549 cells. CHEPS inhibited tumour growth as measured by size and weight (Figure 7A, B). However, there was no significant weight loss; on the contrary, a slight increase in body weight was observed compared with controls (Figure 7C). H&E staining did not demonstrate any major organ‐related toxicities after CHEPS treatment (Figure 7D). TEM examination of tumour tissues showed that the CHEPS‐treated group displayed more autophagosomes compared with the control group (Figure 7E), implying that CHEPS induced tumour cell autophagy in vivo. This finding was further confirmed by the detection of increased LC3B‐II and decreased p62 protein by immunohistochemistry (Figure 7F). Immunohistochemistry also demonstrated that CHEPS increased ERK and p38 phosphorylation (Figure 7F), suggesting that CHEPS activated ERK and p38 in tumour tissues in vivo. The lipid peroxidation product malondialdehyde (MDA), a marker of ROS‐mediated injury in vivo, was also increased in tumour tissues upon CHEPS treatment (Figure 7G), indicating that CHEPS induced ROS generation in vivo. These data show that CHEPS exhibits potent anti‐tumour activity with low toxicity in vivo. Further, the mechanisms through which CHEPS inhibits NSCLC cells in vitro may also translate in vivo.

## 4 DISCUSSION

Antarctica is the coldest and driest continent in the world. Antarctic microbes have unique mechanisms and biochemical reactions, such as cold, drought and radiation resistance, allowing them to adapt to the environment. Microbial polysaccharides are an important source of natural anti‐tumour drugs. Therefore, we extracted EPS from the previously isolated microorganism from Antarctica, *C. heimaeyensis* S20, and named it CHEPS. In our preliminary experiments, we found that CHEPS significantly inhibited the growth of a variety of tumour cells, including human epidermal cancer cells (A431), ovarian cancer cells (SKOV‐3), cervical cancer cells (HeLa), breast cancer cells (MDA‐MB‐231) and lung cancer cells (A549) (data not shown). Lung cancer is the leading cause of cancer‐related death worldwide.^1^, ^2^ NSCLC accounts for approximately 80% of lung cancer and has a low survival rate.^3^ Thus, we chose NSCLC for further investigation.

CCK8 analysis and colony formation assay showed that CHEPS effectively inhibited the proliferation of NSCLC cells (A549, NCI‐H1299 and SK‐MES‐1) with little cytotoxicity to normal human embryonic lung fibroblasts (WI‐38 and MRC‐5). It is noteworthy that the cell viability of WI‐38 at 72 h after CHEPS treatment was slightly higher than that at 48 h, which may be due to prolonged drug adaptation (Figure 1). The characteristics of primary lung cells are closer to the real situation. We are also considering cooperating with hospitals in the future to use primary human lung fibroblasts and epithelial cells as normal control cells for drug sensitivity verification after passing the ethical review. Most chemotherapeutics have significant toxicities in normal cells; thus, CHEPS is a novel agent for NSCLC that may have lower toxicity than currently available treatments.

Apoptosis is a programmed mechanism of cell death and is essential for normal development and tissue homeostasis.^44^ Avoidance of apoptosis is an important survival mechanism for tumour cells. A major goal in cancer treatment is to specifically trigger tumour cell apoptosis.^45^ In our study, we found that CHEPS did not induce apoptosis (Figure 2A, B). Caspase‐independent pathways also contribute to cell death. Autophagy, a caspase‐independent pathway, is a promising, novel strategy for enhancing the anti‐tumour efficacy of chemotherapy drugs.^18^, ^22^ Although autophagy as a cell death mechanism has been controversial and remains mechanistically undefined, there is growing evidence that autophagy facilitates cell death in specific contexts.^19^, ^23^, ^46^ Our study showed that CHEPS significantly induced the initiation of autophagy (Figure 2C, D; Figure S1A; Figure 3A, B) and triggered complete autophagic flux (Figure 3C–E) in NSCLC and WI‐38 cells. p62 is an adaptor protein located on the autophagosome, which mediates the connection between LC3 and the ubiquitination substrate, and transports the ubiquitinated protein with the autophagosome to the lysosome for degradation. Interestingly, in our in vitro experiments, p62 expression was not consistently lower than control after CHEPS treatment (Figure 2F), which contradicted the assumption that p62 would be degraded when autophagic flux occurred. However, this does not mean that CHEPS‐induced autophagy is incomplete, because in some special conditions or cell types, a constant level, or an increase in level, of the p62 protein does not mean autophagic flux is inhibited, and a decrease in p62 protein does not indicate the integrity of autophagic flux.^47^ Our results reflect dynamic processes of autophagic flux and p62 degradation. Another reason may be that the extent of p62 mRNA upregulation is higher than that of p62 protein degradation (Figure 2E). In our research, CHEPS induced autophagic cell death in NSCLC cells, whereas the role that autophagic cell death played in CHEPS‐treated WI‐38 was less apparent (Figure 3F). The autophagy induction mechanism of CHEPS in WI‐38 cells may be different from that in NSCLC cells, and the autophagy function may also be different; this deserves further exploration. ShATG5 attenuated CHEPS‐induced autophagy (Figure S1B, C), implying that autophagy triggered by CHEPS is ATG5‐dependent. Accordingly, shATG5 modestly rescued CHEPS‐induced cell death in NSCLC cells (Figure S1D), further proving that CHEPS‐induced autophagy serves as a mediator of death in NSCLC cells.

Cell division is a precisely regulated process. CDK2 regulates progression from the G1 to the S phase, and cyclin B‐CDK1 regulates progression from the G2 to the M phase.^48^ Genotoxic stress upregulates the expression of p53, which works with Rb to downregulate a large number of genes that encode proteins required for the S and G2/M phases, leading to p53‐dependent cell cycle arrest. Genotoxic stress also activates p53‐independent pathways that inhibit CDK1 activity.^49^ p21 is induced by both p53‐dependent and ‐independent mechanisms following stress, and induction of p21 may cause cell cycle arrest.^50^ Our study showed that CHEPS induced S‐ and G2/M‐phase arrest with upregulation of p53 and p21 and downregulation of CDK2, Cyclin B1, Cyclin A2 and CDK1 in NSCLC cells (Figure S5). However, NCI‐H1299 is a p53‐null cell line; therefore, CHEPS‐induced cell cycle arrest may also involve p53‐independent mechanisms. Cell cycle arrest occurred but was less pronounced in WI‐38 cells compared with NSCLC. These data are consistent with the finding that CHEPS effectively inhibited proliferation of NSCLC cells but not WI‐38.

Many signals have been reported to regulate cell autophagy and death.^18^, ^19^ AMPK is an intracellular energy receptor that promotes autophagy when nutrients and energy are scarce. Our research showed that CHEPS activated the AMPK pathway. However, activated AMPK was not involved in CHEPS‐induced autophagic cell death (Figure S3). The MAPK pathway plays a key role in the development and progression of cancer. Accumulating evidence indicates that cell cycle progression and autophagy may be regulated by MAPK signalling.^23^, ^51^, ^52^, ^53^ Here, we found that p38 and ERK were activated by CHEPS and contributed to CHEPS‐induced cell death in NSCLC cells (Figure 4, Figure S2). Further research showed that ERK promoted CHEPS‐induced S‐ and G2/M‐phase arrest (Figure S6A, B). Both p38 and ERK facilitated CHEPS‐induced autophagy in NSCLC cells (Figure 5). However, CHEPS did not significantly induce p38 and ERK activation in WI‐38 cells (Figure 4A, Figure S2A), indicating that CHEPS‐induced autophagy in WI‐38 cells is through other upstream signals; this may be one reason that CHEPS‐induced autophagy does not lead to WI‐38 cell death. Signals upstream of CHEPS‐triggered autophagy in WI‐38 cells remain to be established.

ROS is considered an important upstream molecule in the regulation of cell death and survival in cancer. Moderate production of ROS may provide a pro‐survival signal to cells, but excessive levels of ROS interfere with cellular signalling pathways and cause oxidative damage to cells.^42^ Cancer cells have higher basal ROS levels than their normal counterparts due to increased metabolic stress and proliferative capacity.^28^ Thus, cancer cells may be more sensitive to oxidative stress, which may provide a selective mechanism to induce cell death. The present study showed that CHEPS induced a significant increase in ROS generation in NSCLC cells (Figure S4A, B, Figure 6A). CHEPS‐induced ROS promoted activation of p38 and ERK, cell autophagy, and death, which were verified by NAC treatment (Figure 6B – E). However, NAC did not completely reverse CHEPS‐induced p38 and ERK activation, autophagy or death, implying that there are additional functional targets besides ROS. CHEPS did not induce ROS accumulation in WI‐38 cells (Figure S4A, B), which may be a further reason why CHEPS does not effectively induce WI‐38 cell death. Although many reports indicate that ROS induce cell cycle arrest,^24^, ^54^ CHEPS‐induced ROS had no effect on ERK‐regulated S‐ and G2/M‐phase arrest in our study (Figure S6C, D), indicating that ROS‐induced ERK activation is mainly used to promote autophagy‐dependent cell death and there may be other signalling molecules upstream of ERK to regulate S‐ and G2/M‐phase arrest.

In order to further verify the anti‐tumour effect of CHEPS in vivo, we established a nude mouse model of NSCLC cell xenograft in situ by subcutaneous inoculation of A549 cells. Our results revealed that CHEPS at a dose of 100 mg/kg suppressed orthotopic lung tumour growth (Figure 7A, B), without causing body weight loss and organ toxicity (Figure 7C, D). This is different from some traditional chemotherapeutic drugs such as cisplatin and 5‐fluorouracil, which often show obvious body and organ toxicity, greatly limiting their use. These results suggest the safety and efficacy of CHEPS as a non‐chemosynthetic natural active polysaccharide with anti‐cancer effects in vivo. Consistent with in vitro results, CHEPS‐induced production of ROS, activated p38 and ERK pathways, and triggered autophagy in vivo (Figure 7E – G). The protein level of p62 was decreased in vivo, which is inconsistent with the in vitro results. We suggest that, because the cell environments in vivo and in vitro are different, the dynamic process of autophagy may also differ in these environments.

**Figure 8:**
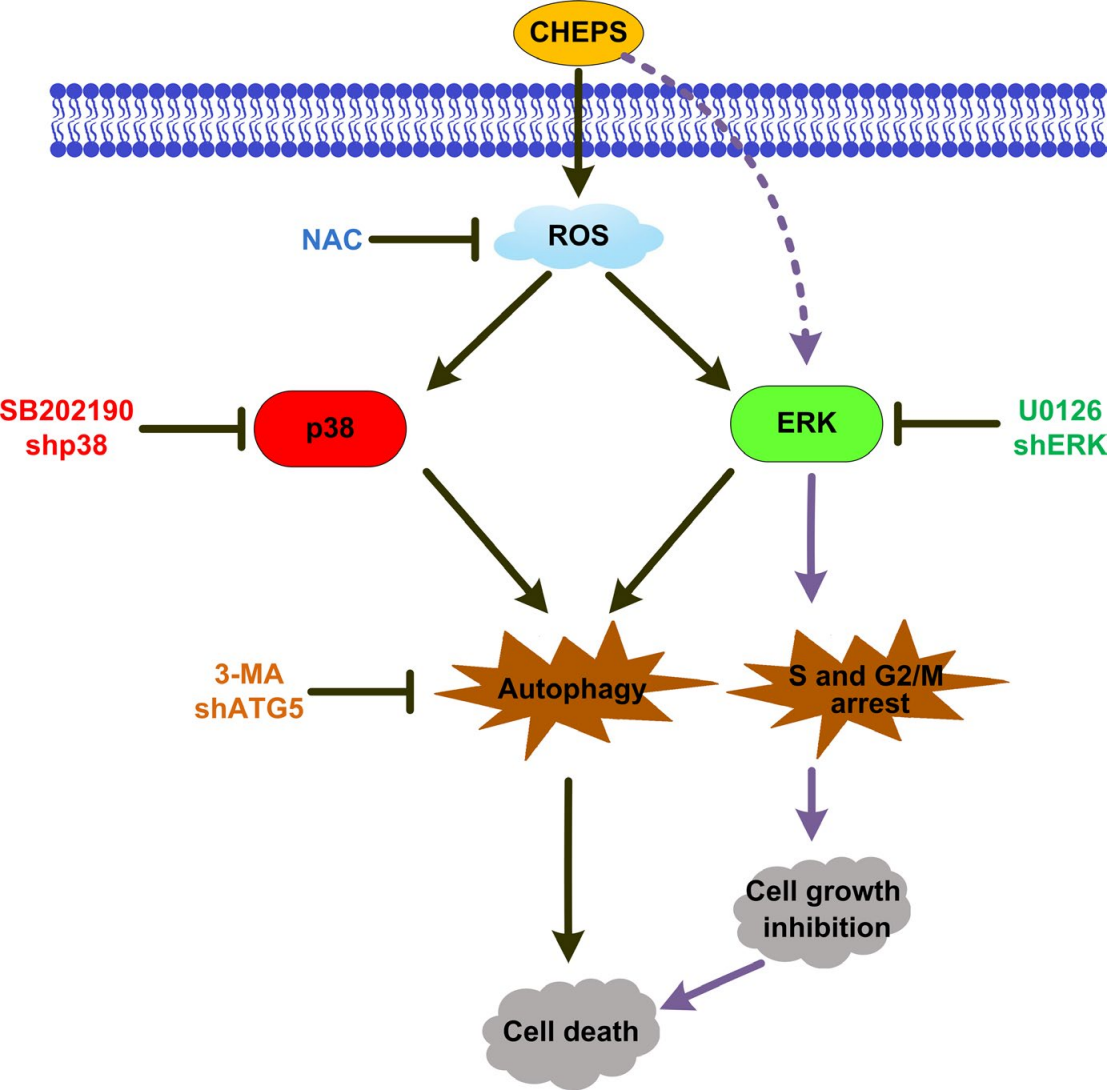
Schematic of the pathways through which CHEPS induces autophagic cell death and S‐ and G2/M‐phase arrest in non‐small cell lung cancer

In conclusion, our study, for the first time, identified the anti‐tumour effects of an exopolysaccharide extract from *C. heimaeyensis* S20 (CHEPS) on NSCLC cells in vitro and in vivo and examined potential molecular mechanisms. We found that CHEPS induces autophagic cell death in NSCLC cells via ROS/p38 and ROS/ERK signalling, and CHEPS induces S‐ and G2/M‐phase arrest via ERK signalling (Figure 8). The cytotoxic effects of CHEPS on normal human embryonic lung fibroblasts in vitro or internal organs of mice in vivo were minimal or none‐existent, indicating that CHEPS may be a novel anti‐tumour drug with great potential as a therapy for NSCLC. Our research also provides a new direction for the exploration of Antarctic microbial resources.

## CONFLICT OF INTERESTS

The authors declare no conflicts of interest.

## AUTHOR CONTRIBUTIONS

Yao Hao designed, conducted, analysed and interpreted the experiments, and wrote the manuscript. Yao Huang and Danqing Lin extracted the exopolysaccharide CHEPS. Jingyi Chen participated in the animal experiments. Jiadai Li did some molecular cloning and qRT‐PCR experiments. Yuncong Yuan and Mingzhen Wang carried out some Western blot experiments. Lingling Han, Xiu Xin and Hailong Wang did some data analysis. Fang Peng and Fang Yu proofread the manuscript. Congyi Zheng designed the experiments and revised the manuscript. Chao Shen designed and supervised the experiments, revised the manuscript and approved the final version to be published.

## Supporting information

Fig S1

Fig S2

Fig S3

Fig S4

Fig S5

Fig S6

Table S1

Supplementary Material

## Data Availability

The authors declare that the data supporting the findings of this study are available from the corresponding author upon reasonable request.
