# Supplementary figures and images for: Exopolysaccharide from Cryptococcus heimaeyensis S20 induces autophagic cell death in non‐small cell lung cancer cells via ROS/p38 and ROS/ERK signalling
Source: Cell Prolif. 2020 Jun 29;53(8):e12869. doi: 10.1111/cpr.12869 (PMC7445402; doi:10.1111/cpr.12869)

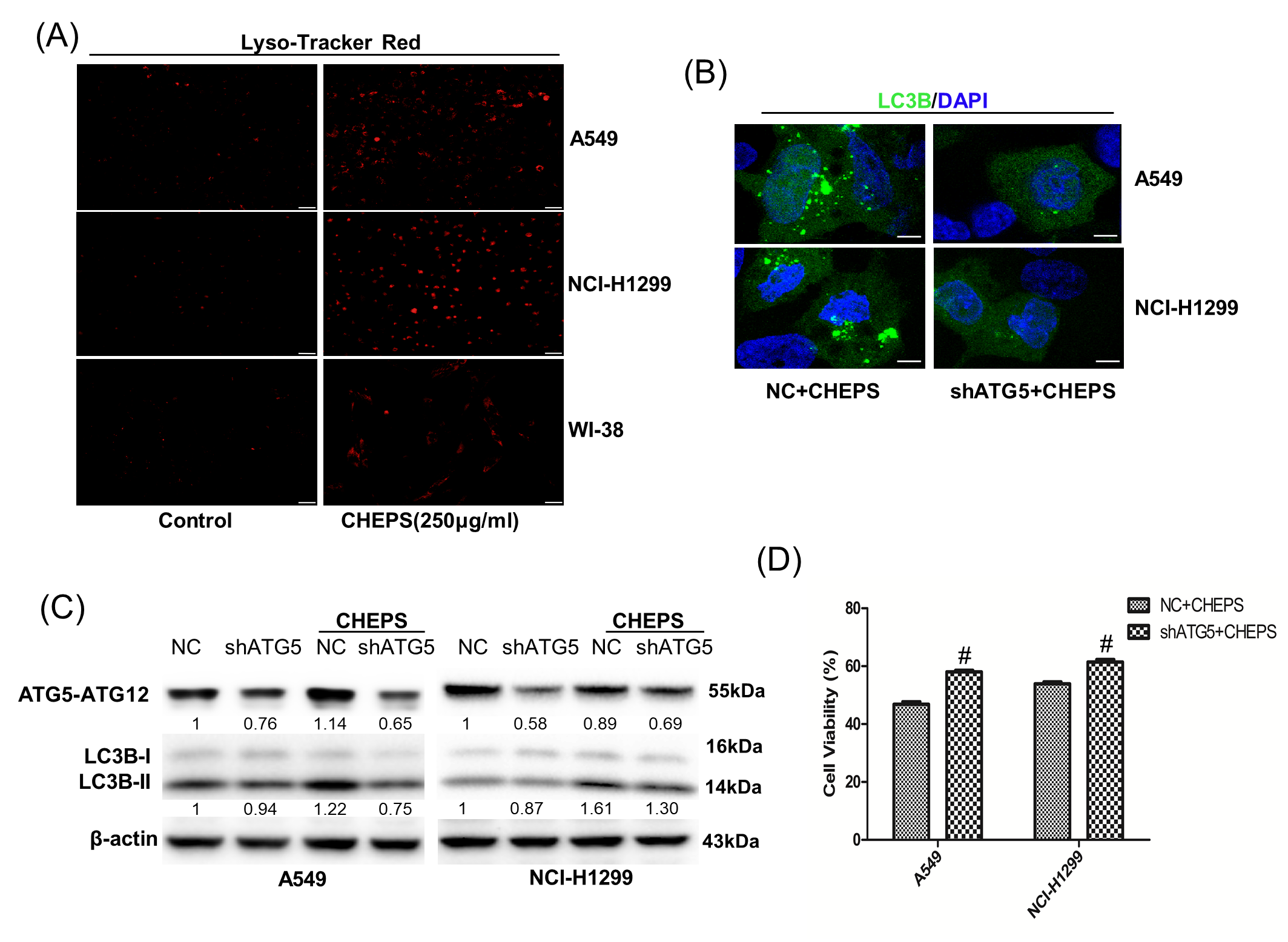

Supplement: Supplementary file 1 — Fig S1 [file CPR-53-e12869-s001.tif]

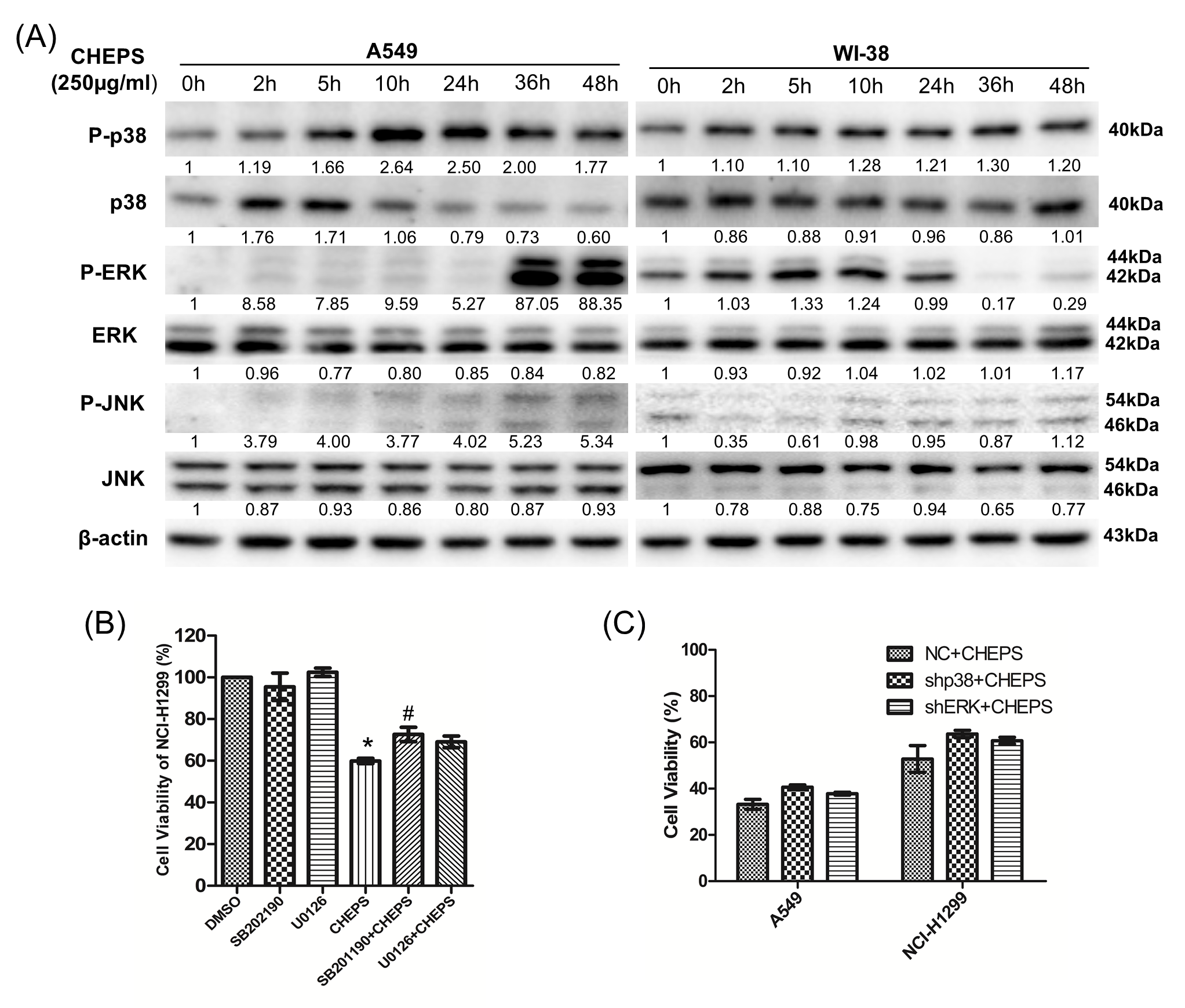

Supplement: Supplementary file 2 — Fig S2 [file CPR-53-e12869-s002.tif]

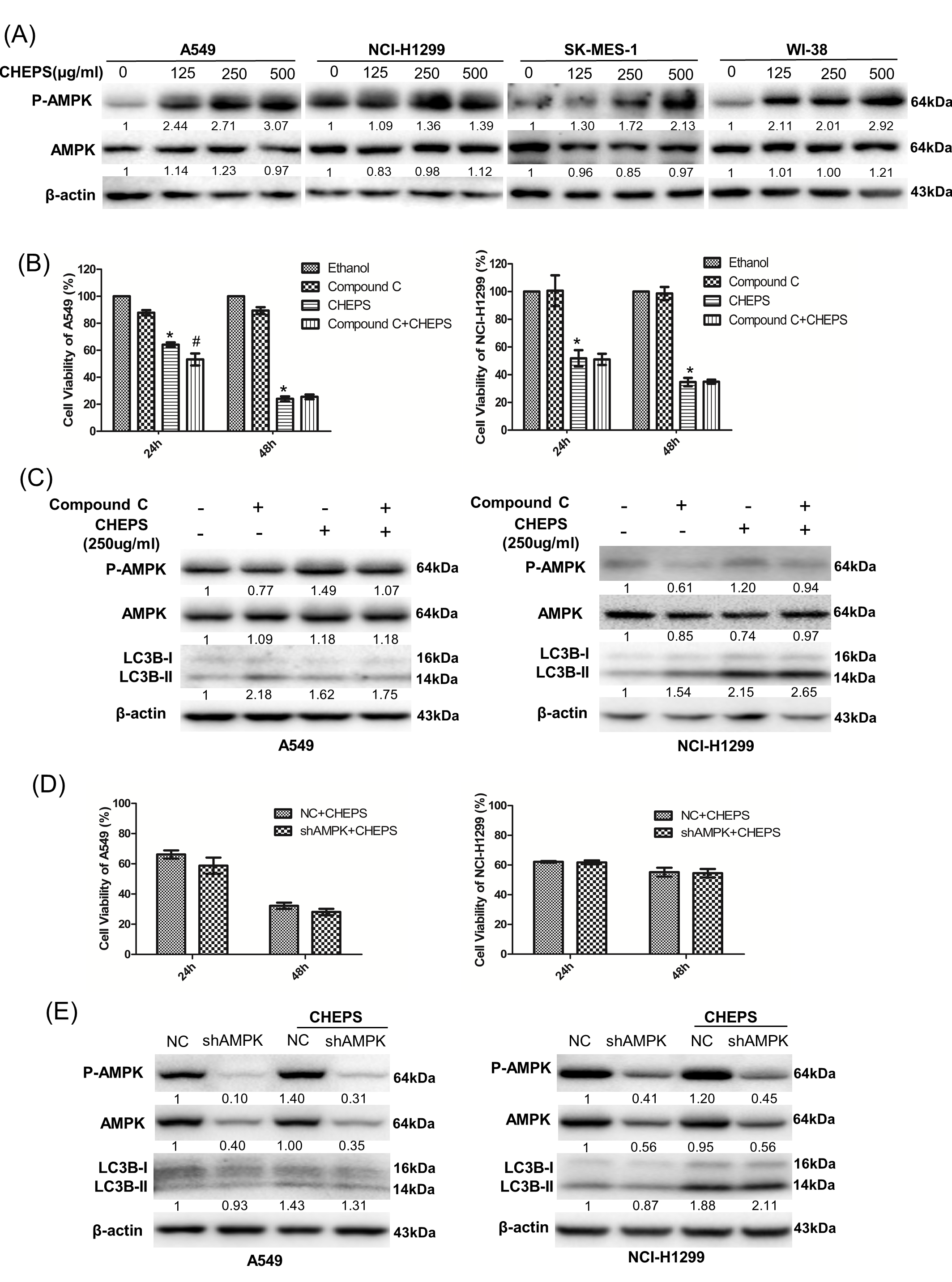

Supplement: Supplementary file 3 — Fig S3 [file CPR-53-e12869-s003.tif]

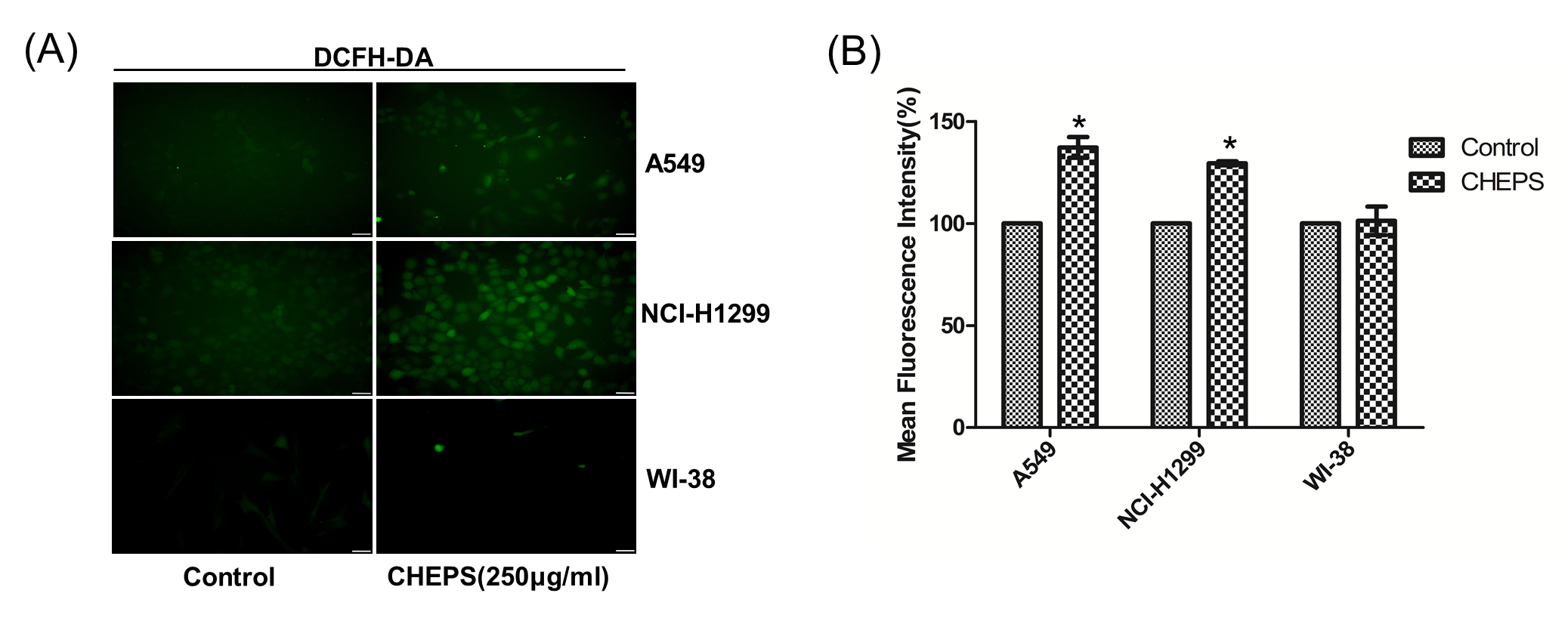

Supplement: Supplementary file 4 — Fig S4 [file CPR-53-e12869-s004.tif]

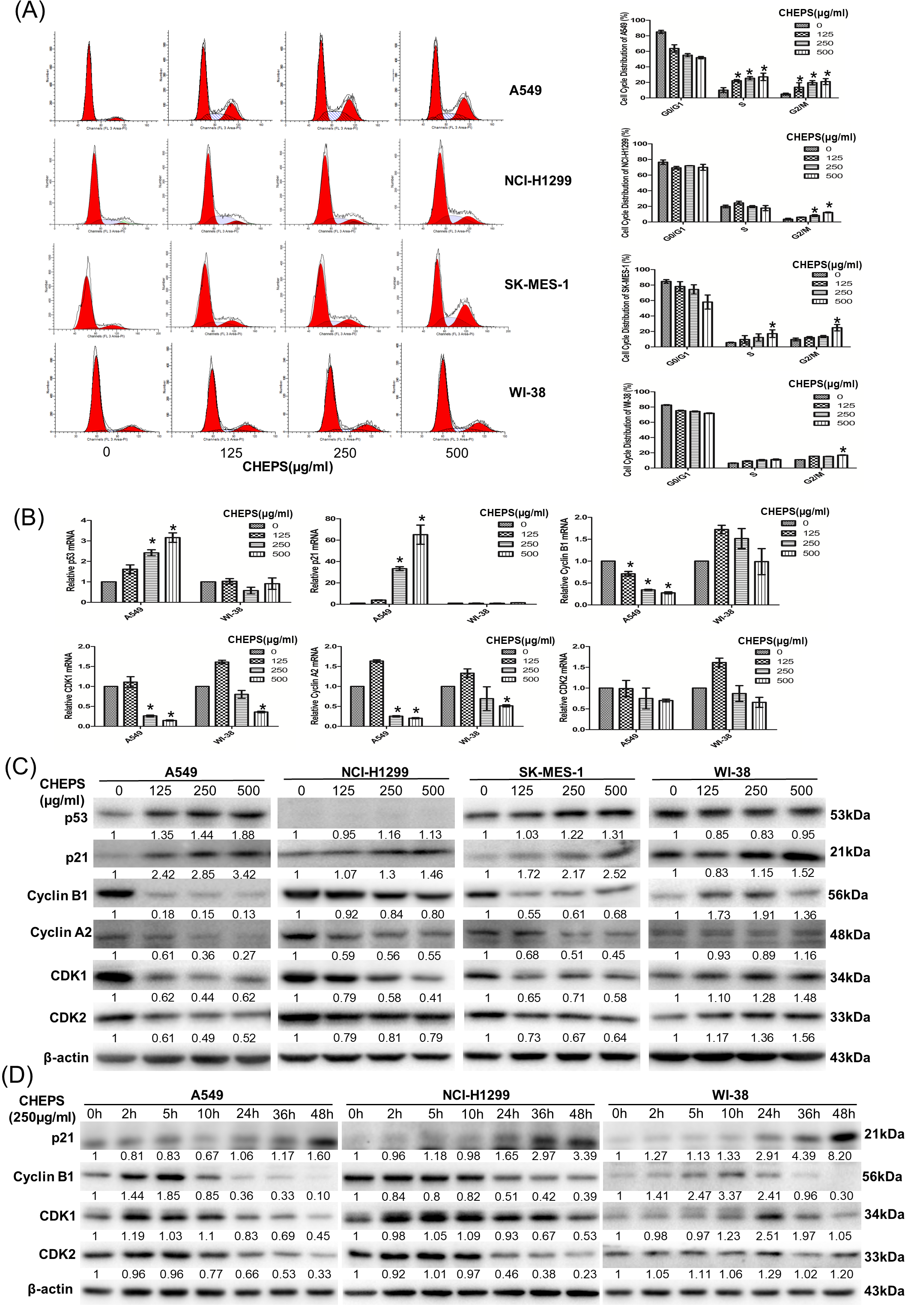

Supplement: Supplementary file 5 — Fig S5 [file CPR-53-e12869-s005.tif]

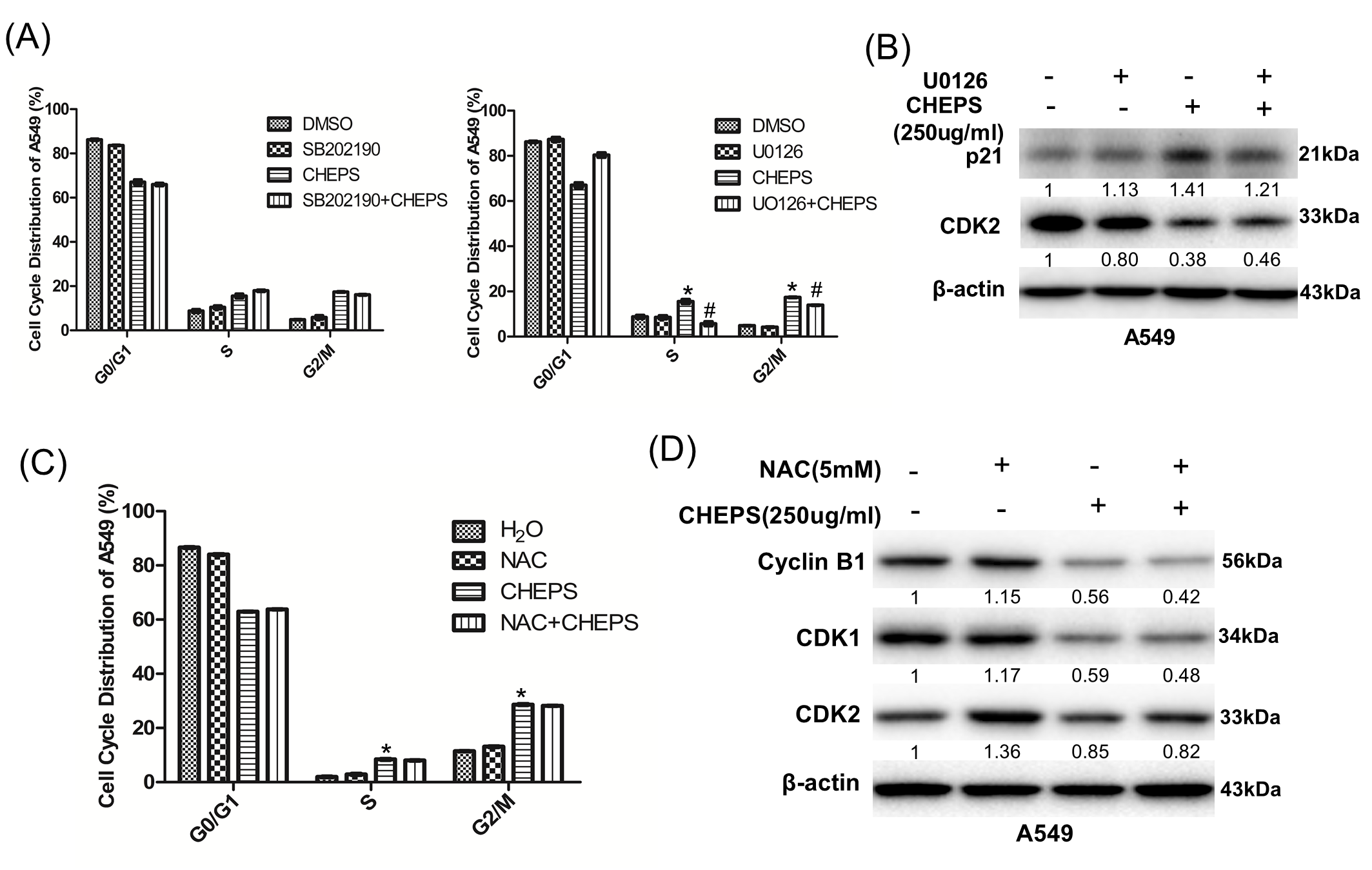

Supplement: Supplementary file 6 — Fig S6 [file CPR-53-e12869-s006.tif]
