# Supplementary material for: Exopolysaccharide from Cryptococcus heimaeyensis S20 induces autophagic cell death in non‐small cell lung cancer cells via ROS/p38 and ROS/ERK signalling
Source: Cell Prolif. 2020 Jun 29;53(8):e12869. doi: 10.1111/cpr.12869 (PMC7445402; doi:10.1111/cpr.12869)
Supplement: Supplementary file 7 — Table S1 [file CPR-53-e12869-s007.docx]

Table S1. qPCR primers

| qPCR primers | Fp（5'to 3'） | Rp（5'to 3'） |
| --- | --- | --- |
| p53 | AGGCCTTGGAACTCAAGGAT | TGAGTCAGGCCCTTCTGTCT |
| p21 | GGGATGAGTTGGGAGGAGG | CGGCGTTTGGAGTGGTAG |
| Cyclin B1 | AGTTATGCAGCACCTGGCTA | GCCACAGCCTTGGCTAAATC |
| Cyclin A2 | CGGTACTGAAGTCCGGGAAC | CATGAATGGTGAACGCAGGC |
| CDK1 | CCCTCCTGGTCAGTACATGG | GCTCTGGCAAGGCCAAAATC |
| CDK2 | TAACACAGAGGGGGCCATCA | GCGAGTCACCATCTCAGCAA |
| ATG5 | GCTTCGAGATGTGTGGTTTGG | CCATTTCAGTGGTGTGCCTTC |
| ATG6 | AATGGTGGCTTTCCTGGACT | TGATGGAATAGGAGCCGCCA |
| ATG7 | GGCACTGCTCTTGAAAACCC | CACTGGCCCCTGAATGAGAG |
| LC3 | TTCGAGAGCAGCATCCAACC | GATTGGTGTGGAGACGCTGA |
| p62 | CACTGGCCCCTGAATGAGAG | TGGCTGTGAGCTGCTCTT |
| GAPDH | CCACTCCTCCACCTTTGAC | ACCCTGTTGCTGTAGCCA |
