# Supplementary material for: Exopolysaccharide from Cryptococcus heimaeyensis S20 induces autophagic cell death in non‐small cell lung cancer cells via ROS/p38 and ROS/ERK signalling
Source: Cell Prolif. 2020 Jun 29;53(8):e12869. doi: 10.1111/cpr.12869 (PMC7445402; doi:10.1111/cpr.12869)
Supplement: Supplementary file 8 — Supplementary Material [file CPR-53-e12869-s008.docx]

**SUPPLEMENTARY FIGURE LEGENDS**

**FIGURE S1.** A, A549, NCI-H1299, and WI-38 cells were stained by Lyso-Tracker Red following treatment with 250 µg/ml CHEPS for 24 h. Red color intensity shows acidic vesicular organelles, indicating lysosomes and autolysosomes. Bar: 50 µm. B, Stable cell lines (A549-pSUPER NC, A549-shATG5, NCI-H1299-pSUPER NC, NCI-H1299-shATG5) were pre-transfected with the pEGFP-LC3B plasmid for 24 h and then treated with CHEPS (250 µg/ml) for 5 h followed by observation under a confocal microscope. Bar: 10 µm. C, Stable cell lines (A549-pSUPER NC, A549-shATG5, NCI-H1299-pSUPER NC, NCI-H1299-shATG5) were treated with or without CHEPS (250 µg /ml) for 5 h. Cell lysates were examined for ATG5 and LC3B expression by western blotting. ATG5 antibody detected the covalent complex of ATG5-ATG12. D, Stable cell lines (A549-pSUPER NC, A549-shATG5, NCI-H1299-pSUPER NC, NCI-H1299-shATG5) were treated with or without CHEPS (250 µg /ml) for 48 h, and cell viability was determined by CCK8 assay. Data represent the mean ± S.D. # P < 0.05 versus NC+CHEPS.

**FIGURE S2.** A, A549 and WI-38 cells were treated with 250 µg/ml CHEPS for different hours. Cell lysates were examined for MAPK-related proteins by western blot analysis. B, SB202190 (2 µM, p38 inhibitor) and U0126 (2 µM, ERK inhibitor) were added to NCI-H1299 cells 2 h before CHEPS (250 µg/ml) treatment. After 24 h, cell viability was determined by CCK8 assay. Data represent the mean ± S.D. from three independent experiments. *P < 0.05 versus DMSO, # P < 0.05 versus CHEPS treatment. C, Stable cell lines (A549-pSUPER NC, A549-shp38, A549-shERK, NCI-H1299-pSUPER NC, NCI-H1299-shp38, H1299-shERK) were treated with 250 µg/ml CHEPS for 48 h. CCK8 assay was used to determine cell viability. Data represent the mean ± S.D. from three independent experiments.

**FIGURE S3** AMPK signaling is induced by CHEPS but not required for CHEPS-induced cell death. A, A549, NCI-H1299, SK-MES-1, and WI-38 cells were treated with CHEPS (0, 125, 250, 500 µg/ml) for 48 h. AMPK signaling proteins were analyzed by western blotting. B, Compound C (2 µM) was added to A549 and NCI-H1299 2 h before CHEPS (250 µg/ml) treatment. After 24 h and 48 h, cell viability was determined by CCK8 assay. Data represent the mean ± S.D. from three independent experiments. *P < 0.05 versus Ethanol, # P < 0.05 versus CHEPS treatment. C, Compound C (2 µM) was added to A549 and NCI-H1299 2 h before CHEPS (250 µg/ml) treatment. After 24 h, cell lysates were examined for P-AMPK and LC3B by western blot analysis. D, Stable cell lines (A549-pSUPER NC, A549-shAMPK, NCI-H1299-pSUPER NC, NCI-H1299-shAMPK) were treated with 250 µg/ml CHEPS for 24 h and 48 h, and cell viability was determined by CCK8 assay. Data represent the mean ± S.D. from three independent experiments. E, Stable cell lines (A549-pSUPER NC, A549-shAMPK, NCI-H1299-pSUPER NC, NCI-H1299-shAMPK) were treated with 250 µg/ml CHEPS for 24 h. Cell lysates were examined for P-AMPK and LC3B by western blot analysis.

**FIGURE S4.** A, A549, NCI-H1299 and WI-38 cells were treated with 250 μg/ml CHEPS for 24 h followed by DCFH-DA staining and observation under a fluorescence microscope. Bar: 50 µm. B, A549, NCI-H1299 and WI-38 cells were treated with 250 μg/ml CHEPS for 24 h. Cells were stained with DCFH-DA and examined by flow cytometry.

**FIGURE S5** CHEPS induces S- and G2/M-phase arrest by regulating cell cycle-related proteins. A, CHEPS induces S- and G2/M-phase arrest. A549, NCI-H1299, SK-MES-1, and WI-38 cells were treated with CHEPS (0, 125, 250, 500 µg/ml) for 48 h and analyzed by flow cytometry. The percentage of cells in each phase is presented as the mean ± S.D. from three independent experiments. B, A549 and WI-38 cells were treated with CHEPS (0, 125, 250, 500 µg/ml) for 48 h. The mRNA levels of cell cycle-regulated genes were analyzed by RT-qPCR. Data represented the mean ± S.D. from three independent experiments. *P < 0.05, significantly different compared with CHEPS (0µg/ml). C, A549, NCI-H1299, SK-MES-1, and WI-38 cells were treated with CHEPS (0, 125, 250, 500 µg/ml) for 48 h. The expression levels of cell cycle-regulated proteins were analyzed by western blot. D, A549, NCI-H1299, and WI-38 cells were treated with CHEPS (250 µg/ml) for different hours. The expression levels of cell cycle-regulated proteins were analyzed by western blot.

**FIGURE S6** A, SB202190 (2 µM) and U0126 (2 µM) were added to A549 2 h before CHEPS (250 µg/ml) treatment. After 48 h, cells were collected and analyzed by flow cytometry. Cell cycle distribution is presented as the mean percentage of cells per phase ± S.D. from three independent experiments. *P < 0.05 versus DMSO, # P < 0.05 versus CHEPS treatment. B, U0126 (2 µM) was added to A549 2 h before CHEPS (250 µg/ml) treatment. After 48 h, cell lysates were collected, and expression of cell cycle-regulated proteins were analyzed by western blot. C, After incubation with NAC (5 mM) for 2 h, A549 cells were treated with 250 µg/ml CHEPS with or without NAC for another 24 h. Cells were collected and analyzed by flow cytometry. Cell cycle distribution is presented as the mean percentage of cells per phase ± S.D. from three independent experiments. *P < 0.05 versus H_2_O. D, After incubation with NAC (5 mM) for 2 h, A549 cells were treated with 250 µg/ml CHEPS with or without NAC for another 24 h. Cell lysates were examined for expression of cell cycle-regulated proteins by western blotting.

**Table S1.** qPCR primers.
